# Inferring causal metabolic signals that regulate the dynamic TORC1-dependent transcriptome

**DOI:** 10.15252/msb.20145475

**Published:** 2015-04-17

**Authors:** Ana Paula Oliveira, Sotiris Dimopoulos, Alberto Giovanni Busetto, Stefan Christen, Reinhard Dechant, Laura Falter, Morteza Haghir Chehreghani, Szymon Jozefczuk, Christina Ludwig, Florian Rudroff, Juliane Caroline Schulz, Asier González, Alexandre Soulard, Daniele Stracka, Ruedi Aebersold, Joachim M Buhmann, Michael N Hall, Matthias Peter, Uwe Sauer, Jörg Stelling

**Affiliations:** 1 Department of Biology, Institute of Molecular Systems Biology, ETH ZurichZurich, Switzerland; 2 Department of Biosystems Science and Engineering and SIB Swiss Institute of Bioinformatics, ETH ZurichBasel, Switzerland; 3 Department of Computer Science, ETH ZurichZurich, Switzerland; 4 Department of Biology, Institute of Biochemistry, ETH ZurichZurich, Switzerland; 5 Biozentrum, University of BaselBasel, Switzerland; 6 UMR5240 MAP, Université Lyon 1Villeurbanne, France; 7 Faculty of Science, University of ZurichZurich, Switzerland

**Keywords:** causal inference, network motifs, nutrient signaling, target of rapamycin pathway

## Abstract

Cells react to nutritional cues in changing environments via the integrated action of signaling, transcriptional, and metabolic networks. Mechanistic insight into signaling processes is often complicated because ubiquitous feedback loops obscure causal relationships. Consequently, the endogenous inputs of many nutrient signaling pathways remain unknown. Recent advances for system-wide experimental data generation have facilitated the quantification of signaling systems, but the integration of multi-level dynamic data remains challenging. Here, we co-designed dynamic experiments and a probabilistic, model-based method to infer causal relationships between metabolism, signaling, and gene regulation. We analyzed the dynamic regulation of nitrogen metabolism by the target of rapamycin complex 1 (TORC1) pathway in budding yeast. Dynamic transcriptomic, proteomic, and metabolomic measurements along shifts in nitrogen quality yielded a consistent dataset that demonstrated extensive re-wiring of cellular networks during adaptation. Our inference method identified putative downstream targets of TORC1 and putative metabolic inputs of TORC1, including the hypothesized glutamine signal. The work provides a basis for further mechanistic studies of nitrogen metabolism and a general computational framework to study cellular processes.

## Introduction

The comprehensive elucidation of the multi-level, dynamic molecular networks that underlie the processes of living cells requires the generation of dynamic data at different levels of biological organization, the integration of complementary data types, and the incorporation of prior knowledge (Ideker *et al*, 2011; Bar-Joseph *et al*, 2012). Currently large-scale, dynamic experimental datasets are still scarce, but it can be anticipated, based on rapidly advancing technology, that such data will become abundantly available in the near future. Consequently, the primary challenges for the comprehensive analysis of dynamic molecular networks will shift from data generation toward data and knowledge integration. At present, most molecular network analyses consider a single data type such as transcript, protein, or metabolite abundances. This precludes the multi-level, integrated analysis of processes, for example, the interplay between signaling and transcriptional networks. Further, the few studies that have integrated different data types, for example, interacting metabolic and transcriptional networks (Zhu *et al*, 2012), almost always inferred static networks even though the ubiquitous presence of feedback loops suggests that dynamic resolution is required to infer causal relationships between network components (Bar-Joseph *et al*, 2012). Finally, even with prior knowledge integration by static maps (e.g. of transcriptome data onto known transcription factor networks) or by large-scale mathematical models (e.g. representations of metabolic constraints), the inference of causal networks is a largely unsolved problem (Orth & Palsson, 2010; Ideker *et al*, 2011; Bar-Joseph *et al*, 2012).

These limitations particularly affect studies of metabolism (Fernie & Stitt, 2012), because metabolism both integrates external stimuli and generates internal signals that orchestrate cellular adaptation, for example by regulating the activity of kinases (Wilson & Roach, 2002; Dechant & Peter, 2008), transcription factors (Sellick & Reece, 2005), and metabolic enzymes (Link *et al*, 2013) in response to metabolite abundances. For example, the activity of several transcription factors is feedback-regulated via the direct binding of intermediates of metabolic pathways that are under their control (Sellick & Reece, 2005; Pinson *et al*, 2009). Statistical descriptions in most existing dynamic multi-omics studies associate metabolite changes only qualitatively to the consequences of transcriptional and translational regulation of metabolism (Kresnowati *et al*, 2006; Dikicioglu *et al*, 2012). Functional relations between genes and metabolites were recently inferred with Bayesian networks from metabolome, transcriptome, and other datasets (Bradley *et al*, 2009; Zhu *et al*, 2012). However, simple Bayesian networks cannot consider feedback and are therefore unable to describe the exact nature of most metabolic signaling systems. Consequently, despite the frequent observations that nutritional stimuli modulate signaling and transcriptional networks, the exact nature of most metabolic signals is elusive and their identification remains a challenge (Wilson & Roach, 2002; Dechant & Peter, 2008; Watson *et al*, 2013).

To infer causal relationships between potential metabolic signals and the kinases and/or transcription factors they regulate, we co-designed a perturbation matrix for the generation of dynamic multi-omics datasets and a probabilistic, model-based analysis method that systematically incorporates prior knowledge. We focused on nitrogen (N) metabolism of *Saccharomyces cerevisiae* where general and specific mechanisms sense and react to the quality of the available N-source (Magasanik & Kaiser, 2002; Ljungdahl & Daignan-Fornier, 2012). Cellular responses to N-source quality are tightly regulated via the TOR kinase complexes (Crespo *et al*, 2002; Loewith & Hall, 2011), and their downstream effects are relatively well studied (Rohde *et al*, 2008; Loewith & Hall, 2011). For example, yeast uses glutamine preferentially over the alternative N-source proline, partly by nitrogen catabolite repression (NCR) of proline utilization (Hofman-Bang, 1999; Magasanik & Kaiser, 2002). The response to rapamycin-induced inhibition of the TOR complex 1 (TORC1) resembles the response to less-preferred N-sources (Smets *et al*, 2010; Loewith & Hall, 2011), namely inhibition of ribosomal biogenesis and induction of autophagy, pseudohyphal growth, and NCR-controlled pathways. At the molecular level, TORC1 is known to directly target the kinase Sch9 and the protein phosphatase 2A complex (PP2Ac) and to modulate the phosphorylation state of several other kinases and transcription factors (Smets *et al*, 2010; Loewith & Hall, 2011). Conversely, while intracellular glutamine and leucine concentrations have been suggested as upstream endogenous signal(s) to TORC1, the exact mechanisms are unclear (Crespo *et al*, 2002; Gaubitz & Loewith, 2012). Here, we performed dynamic multi-level omics measurements of yeast cells perturbed by the modulation of the quality of the N-source and by chemical inhibition of TORC1. Our metabolome, transcriptome, and TOR-related sub-proteome data captured the dynamics of the underlying regulation mechanisms, and our probabilistic, model-based inference method identified putative metabolic signals up- and downstream of TORC1, including the suggested glutamine signal, and novel, enzymatic targets of TORC1 signaling.

## Results

### A comprehensive and consistent dataset for dynamic shifts between nitrogen sources

**Figure 1:**
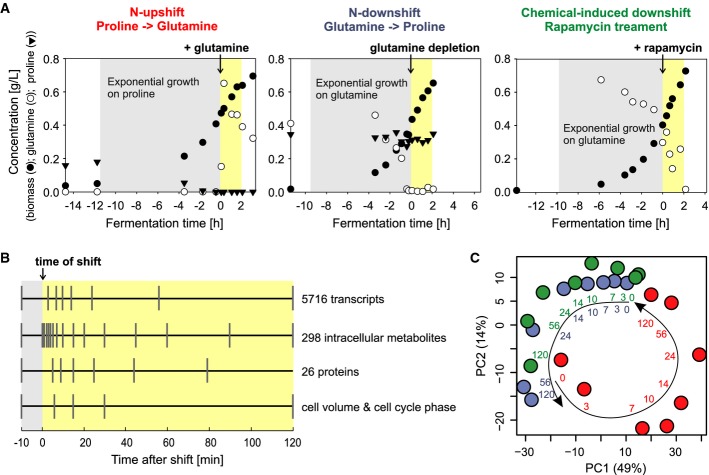
Experimental design and data consistency

To elucidate the dynamic interplay between the signaling, transcriptional, and metabolic networks controlling the cellular response to the quality of the external N-source, we subjected *S. cerevisiae* YSBN6 wild-type cells (Canelas *et al*, 2010) to nutritional upshift and downshifts that were each done as fully independent biological triplicate experiments. Dynamic shifts in well-controlled bioreactor batch cultures with glucose minimal medium were induced by adding glutamine to yeast growing exponentially on the poor N-source proline (upshift) or by glutamine depletion in yeast growing on glutamine plus proline (downshift) (Fig1A, Supplementary Tables S1 and S2). In the upshift, proline was nearly depleted at the time of the shift, but it still sustained exponential growth, and added glutamine was almost immediately taken up. In the downshift, exponentially growing cells exclusively consumed glutamine until its complete depletion, as expected from NCR (Hofman-Bang, 1999; Magasanik & Kaiser, 2002). Additionally, we induced a downshift chemically by adding rapamycin to a culture growing exponentially on glutamine as the sole N-source, thereby bypassing the natural nitrogen quality signal by inhibiting TORC1 directly (Loewith & Hall, 2011) (Fig1A). Expectedly, TORC1 activity increased rapidly during the upshift and decreased during both downshifts, as measured by the phosphorylation status of Sch9 (Urban *et al*, 2007; Loewith & Hall, 2011) (Supplementary Fig S1, Supplementary Table S3). Likewise, cell volume and percentage of cells in the G1 phase of the cell cycle matched the expectations (Loewith *et al*, 2002) (Supplementary Fig S2, Supplementary Table S4).

Biomass evolution (filled circles) and extracellular concentrations of glutamine (open circles) and proline (triangles) for the three dynamic shifts. Fermentation time is scaled relative to the time of shift. Grey areas mark the exponential growth phase prior to the shift. Yellow areas mark the 2 h following the shifts during which samples were taken. The maximum specific rates of steady-state growth on proline and glutamine plus proline were 0.20 ± 0.03/h (*N* = 3, SD) and 0.36 ± 0.01/h (*N* = 6, SD), respectively. See also Supplementary Tables S1 and S2.

Sampling times for the different measurements relative to the time of shift, taken in independent biological triplicates. The sample −10 min represents the steady-state cellular status preceding the shifts and is henceforth referred to as time 0. See also Supplementary Tables S4, S5, S6 and S7.

Scores plot of the first two principal components (PC1, 2; variance captured by each component in parentheses; PC3 explains 10% of the variance) for the transcripts of the 909 metabolic genes from all time point samples across the three shift experiments (red: N-upshift; blue: N-downshift; green: rapamycin-induced downshift; numbers near arrows: minutes after shift). For transcript data, see Supplementary Table S5.

To capture the regulatory events triggering or reflecting cellular adaptation to the shifts, we quantified the intracellular abundances of 5,716 transcripts by expression array analysis, 20 TOR-related signaling proteins and six enzymes of glutamine–glutamate metabolism by targeted proteomics (Picotti *et al*, 2013), and 42 metabolites by targeted LC-MS/MS (Buescher *et al*, 2010) (Supplementary Tables S5, S6 and S7). A further 256 metabolites were analyzed by untargeted semi-quantitative flow injection time-of-flight MS (Fuhrer *et al*, 2011) (Supplementary Table S7). To minimize biological variability, all samples for the different omics analyses were withdrawn from one bioreactor culture in three independent biological replicates, at a temporal coverage adapted to the expected dynamics of metabolite, transcript, and protein responses (Buescher *et al*, 2012) (Fig1B). Since all 20 TOR-related signaling proteins showed constant abundances during the shifts (Supplementary Fig S3), we assessed the consequences of signaling at the metabolic and transcription level.

Altogether, 3,203, 1,585, and 2,104 transcript abundances changed by more than two-fold (maximum observed fold-change across the time course) in the upshift, the nutritional, and the rapamycin-induced downshift, respectively. We tested the consistency of our dataset across experiments by principal component analysis of transcript dynamics for the 909 metabolic enzymes (Herrgard *et al*, 2008). The first two principal components, which capture ∼60% of the variance, indicated that the cellular states at the start and end points of up- and downshifts were in close vicinity (Fig1C; see Supplementary Fig S4 for all genes). Also, transcript responses of the nutritional and rapamycin-induced downshifts followed each other closely, confirming that rapamycin addition mimics a nitrogen downshift to proline. We conclude that our dynamic shift experiments were complementary and consistent and therefore suitable to address two specific questions: (i) what is the extent of global rewiring of yeast metabolism, and (ii) how are metabolic signals related to TORC1 signaling as inputs or outputs.

### Extensive dynamic rewiring of metabolism via multiple mechanisms

**Figure 2:**
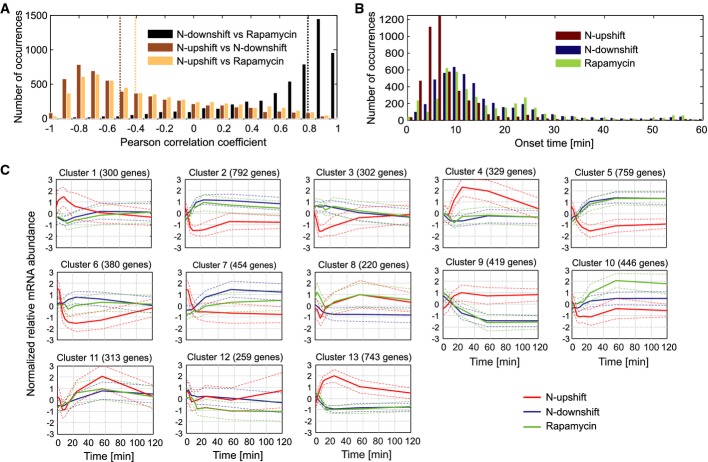
Comparison of transcriptome profiles across the three shifts

The extent of transcriptional responses during the shift experiments indicated a large-scale reorganization of metabolism during adaptation, reminiscent of the global re-wiring during bacterial nutrient source transitions (Buescher *et al*, 2012). Despite the consistency across the three shifts, the broad distributions of transcript correlations (Fig2A) and response timings (Fig2B) indicated rather heterogeneous responses of individual transcripts. Average onset times [times for reaching half of the maximum changes in transcript abundance according to an impulse model (Chechik *et al*, 2008; Chechik & Koller, 2009; Supplementary Table S5)] were around 7 min in the upshift and wider, bimodally distributed in the two downshifts with peaks at around 10 and 25 min. These data, furthermore, indicate that transcriptional responses were highly similar between nutrient downshift and rapamycin perturbation also at the level of individual transcripts, which is a prerequisite for inferring causal relations between metabolites and transcripts based on all pairwise correlations (see below for the corresponding computational method).

Distribution of Pearson's correlation coefficients for every pair of transcripts across all possible pairs of shifts. Dashed lines correspond to the median value for each distribution. See also Supplementary Table S5.

Distribution of onset times (time to reach half of the maximum gene expression change) per shift.

Average transcriptional responses (normalized to the steady-state sample of the N-downshift) for all 13 clusters. Visualization based on the linear interpolated response of the cluster averages. Dashed lines represent ± SD.

The transcript responses across the three shifts distributed over 13 clusters (Fig2C) according to a robust method for the validation of cluster assignments based on maximization of information content (Chehreghani *et al*, 2012). To focus on TORC1-regulated genes, we analyzed clusters 2, 5, 7, 9, 10, and 13, which showed similar responses in the two downshifts, and the opposite behavior in the upshift (Fig2C). Clusters 2, 5, 7, and 10 included most of the known NCR-sensitive genes (Hofman-Bang, 1999) and targets of the retrograde response pathway (Magasanik & Kaiser, 2002) (Supplementary Table S8). They were also over-represented in the gene ontology process terms endocytosis, pseudohyphal growth, mitochondrion organization, and cellular respiration, all of which are induced upon TORC1 inactivation (Loewith & Hall, 2011). The opposite response—induced upshift and repressed downshift expression—was found in clusters 9 and 13 that included nearly all ribosomal protein and ribosome biogenesis genes, known targets of TORC1-regulated transcription factors such as Sfp1 and Fhl1-Ifh1 (Smets *et al*, 2010; Loewith & Hall, 2011). The overall processes captured in the six clusters agreed with previous transcriptional studies in rapamycin-treated yeast (Smets *et al*, 2010; Loewith & Hall, 2011). Documented targets of Gcn4 (Teixeira *et al*, 2006), the general amino acid transcriptional regulator, were over-represented in clusters 1, 8, 9, and 10. Similar responses of cluster 8 transcripts to nutrient upshift and rapamycin treatment indicate additional, TORC1-independent transcriptional regulation.

**Figure 3:**
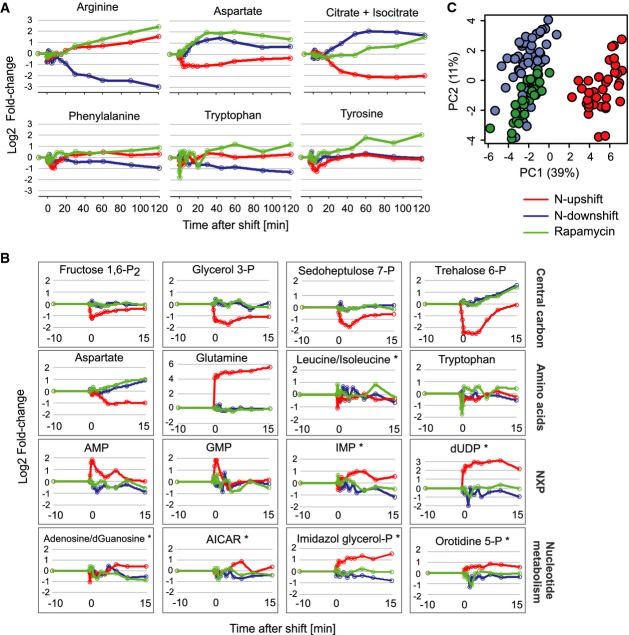
Metabolite profiles across the three shifts

The long-term metabolite response approached a new pseudo steady state within 1 h of perturbation onset (Fig3A and Supplementary Table S7). Specifically, aspartate and citrate/isocitrate levels showed similar long-term trends in the two downshifts and the opposite response in the upshift. The long-term responses of arginine and aromatic amino acids levels were similar between upshift and rapamycin addition, consistent with the starvation-like transcriptional response of their biosynthesis genes in cluster 8 (Fig2C). The immediate metabolite changes within the first 10 min precede transcriptional regulation and reflect readjustments of reaction equilibrium, post-translational, or allosteric enzyme regulation. Within the first minutes, several metabolite abundances changed in the upshift but only very few in the two downshifts (Fig3B). The most pronounced responses in the upshift were the instantaneous increase in intracellular glutamine and the decrease in trehalose 6-phosphate (T6P) concentration, accompanied by transient decreases of metabolite concentrations in upper glycolysis and pentose phosphate pathway, and transient increases of AMP, ADP, GMP, and GDP (Fig3B). In contrast to the glutamine upshift response in *Escherichia coli* (Doucette *et al*, 2011), we observed no changes in glutamate, alpha-ketoglutarate, and other TCA cycle intermediates (Supplementary Fig S5). The similar but not identical early metabolite changes in the two downshifts (Fig3C) were few, subtle, and less dynamic (Fig3B). Since extracellular glutamine is in excess during the rapamycin downshift, early metabolite changes in common with the nutritional downshift may reflect post-translational modifications induced by TORC1 inactivation, while changes specific to the nutritional downshift may be independent or upstream of TORC1. Overall, multiple level adjustments were observed that are non-trivial to explain, that is, they are not just consequences of transcriptional regulation.

Profiles of the metabolites referred to in the text over the 120 min after the shift, for one representative experiment per shift. Fold-changes refer to absolute concentrations normalized to the steady state of the respective shift. Solid lines are the linear interpolated responses, used here for purpose of visualization. See also Supplementary Table S7.

Example of metabolite responses within the first 15 min after the shift for metabolites within central carbon, amino acids, nucleotide mono- and di-phosphates (NXP), and other nucleotide metabolism intermediates. Data for one representative experiment per shift are shown as in (A). Semi-quantitative metabolite concentrations from untargeted FIA-QTOF-MS are marked with *. AICAR: 5-amino-1-(5-phospho-D-ribosyl)imidazole-4-carboxamide; imidazole glycerol-P: D-erythro-1-(imidazole-4-yl)glycerol 3-phosphate.

Principal component analysis of metabolite responses during the first 10 min after the shifts. PC3 explains 9% of the variance.

### A computational method for assigning metabolic signals to network motifs

To identify the metabolic signals that modulate transcriptional responses, and to find how such regulation relates to TORC1 activity, existing computational methods are not sufficient because they do not systematically integrate dynamic metabolomics and transcriptomic data with prior knowledge. For example, current Bayesian integration of transcriptomic and metabolomics data elucidates functional, but not causal relations between the two data types (Bradley *et al*, 2009). By exploiting metabolic network structures as prior knowledge, Chechik *et al* (2008) defined “activity motifs”, patterns such as ordered transcriptional activation along a metabolic pathway, but they only integrated gene expression data. Here, we developed a probabilistic, integrative framework that infers causal relations from heterogeneous data types and exploits prior knowledge on networks and biological mechanisms. To apply the framework to the specific metabolic system of this study, we formalized prior knowledge as prototypic interactions between signaling, metabolism, and gene expression and as generic interaction mechanisms. This is exemplified by a situation where, if a metabolite controls transcription factor activity, a changing metabolite concentration should change the rate of gene expression at a later time, which makes the approach inherently causal.

**Figure 4:**
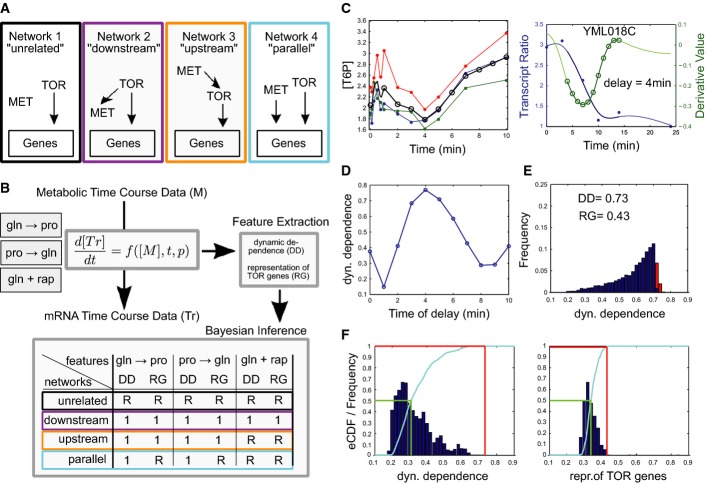
Computational framework for motif assignment

Specifically, our framework classifies the metabolic signals into four network motifs that reflect different modalities of metabolite-TORC1-transcriptional regulation interactions (Fig4A; see Materials and Methods and Supplementary Text S1 for a detailed rationale of the approach). The “unrelated” network motif accounts for metabolite changes unrelated to the transcriptional responses, the “downstream” motif for metabolite responses that are consequences of post-translational enzyme regulations downstream of TORC1, the “upstream” network for metabolites that are potential signals upstream of TORC1, and the “parallel” motif for metabolite responses that modulate transcriptional responses independent of TORC1. The framework uses the early metabolic dynamics and the genome-wide transcript dynamics across the three shift experiments for classification (Fig4B). We assume that an unknown dynamic network connects each metabolic response and the transcript changes over time. At this point, no further assumptions or prior knowledge on the dynamic network and its characteristics are used (see also Supplementary Text S1). From the experimental data, we extract features that approximate: (i) how consistent the observed dynamics are with a causal relation between metabolites and gene expression (“dynamic dependence”; DD), and (ii) how strongly each metabolite is associated with genes that are direct targets of transcription factors regulated by TORC1 (“representation of TOR genes”; RG; see Materials and Methods for details). We use the feature values for each experimental condition to estimate probabilities of metabolite assignments to the network motifs via Bayesian inference (Kass & Raftery, 1995) as explained in detail in Materials and Methods.

Schematic diagrams of the four network motifs (“MET”: metabolite; “TOR”: TORC1 complex; “Genes”: whole genome of *Saccharomyces cerevisiae*). Arrows denote direct causal interactions.

Method overview. For each metabolite monitored under the three experimental conditions (small boxes, upper left), we extract features (grey box, upper right; feature values range between 0 and 1) assuming that the measured quantities are connected by an unknown dynamic network in which metabolite concentrations ([*M*]) control transcript ([*Tr*]) dynamics. The middle grey box illustrates such a causal relation in the form of an ordinary differential equation with an unknown functional relation *f*(·) that depends on time *t* and unknown parameters *p*. Bayesian inference (bottom grey box) is then used to compute the probability of each metabolite being assigned to each network motif characterized by a set of “prototypic” feature values across the three experimental conditions (“R”: representative of artificial, random metabolic time-course data).

Example time-course data from rapamycin downshift. Filled circles represent experimental data points, and open circles denote interpolated data points used for the computation of dynamic dependence. Left: time evolution of T6P (black: linear interpolation from the experimental data; colors: experimental repeats). Right: time evolution of the normalized mRNA level of YML018C (blue line, spline interpolation from the experimental data), and of its derivative values (green line). The thick part of the curves correspond to a 10-min interval with a 4-min time delay used in conjunction with the metabolic data (left) to compute the dynamic dependence for this particular delay.

Dynamic dependence values between T6P and the derivative of YML018C for all delayed versions of the transcript (from 0 to 10 min).

Distribution of dynamic dependence values of T6P with all genes. The top 10% of the distribution (in red) define the significant gene set associated with T6P.

Computation of likelihood values for Bayesian inference of T6P for the “dynamic dependence” feature (left) and “representation of TOR genes” feature (right) computed in the rapamycin downshift. The distribution of the features’ values from artificial, random metabolic time-course data is shown in blue (scaled values), and the corresponding empirical cumulative density function (eCDF) is shown in cyan. The green line denotes the “R” value of (B) and corresponds to the median of the random distributions. Red lines denote the feature values of T6P as shown in (E), used to compute the likelihood value for each network.

Formally, we defined prototypic values for each motif by combining the expected biological events in the three experimental conditions (Fig4B, bottom; see also Supplementary Text S1). A metabolic signal that acts upstream of TORC1 in the two nutritional shifts should elicit a TORC1-dependent transcriptional response. Consequently, the “upstream” network's prototypic feature values for DD and RG are maximal in the two nutrient shifts. However, the metabolic signal is bypassed during rapamycin-induced TORC1 inactivation such that the feature values should not be statistically distinguishable from those of a random response (Fig4B). Similarly, all the “downstream” network's prototypic feature values are maximal for all three experiments because TORC1 should control the same gene sets transcriptionally, and induce metabolite changes post-translationally in all three conditions. For a metabolite to regulate transcription in a TORC1-independent manner (“parallel network”), DD should be high in the two nutrient shifts, but essentially random upon rapamycin treatment (because rapamycin affects only TORC1 activity, not the metabolite concentration), and RG needs to be random in all experiments. Finally, when all feature values of a metabolite are random for all three experiments, we postulate that metabolic and transcriptional changes are not related.

To assign the measured metabolites to the four motifs, we considered every pair of metabolite and transcript in our dataset (Fig4C). Specifically, we computed the dynamic dependence (see Materials and Methods) between every metabolic trajectory and the rate of change of every transcript (Fig4C, right, for YML018C). Signaling metabolite changes should precede their effects on transcriptional regulation, and we allowed for variable delays between 0 and 10 min in 1-min increments for all transcript responses (Fig4C, right). Hence, for every metabolite–transcript pair in every experimental condition, we computed 11 DD values (Fig4D). The maximum values of every pair defined a DD distribution for each metabolite in each experimental condition. For every metabolite, we selected the gene set consisting of 10% of the transcripts with the best DD to calculate the final (average) dynamic dependence (Fig4E) and then determined the fraction of genes known to be targeted by TORC1-dependent transcription factors. In the Bayesian inference framework, these feature values were compared to the distribution of random metabolic data (Fig4F, see Materials and Methods) to estimate network assignment probabilities. To ensure robust assignments, we varied the assumed level of data noise (four variants), the type of interpolation of the metabolite data (2), the method to compute the dynamic dependence of metabolite–transcript pairs (3), and the calculation of network assignments (2), leading to 48 assignment probabilities. Finally, we assigned each metabolite to a network motif by majority vote (exceeding 50% assignment probability in more than half of the methods; Materials and Methods).

### Computational assignment of metabolites to network motifs

**Figure 5:**
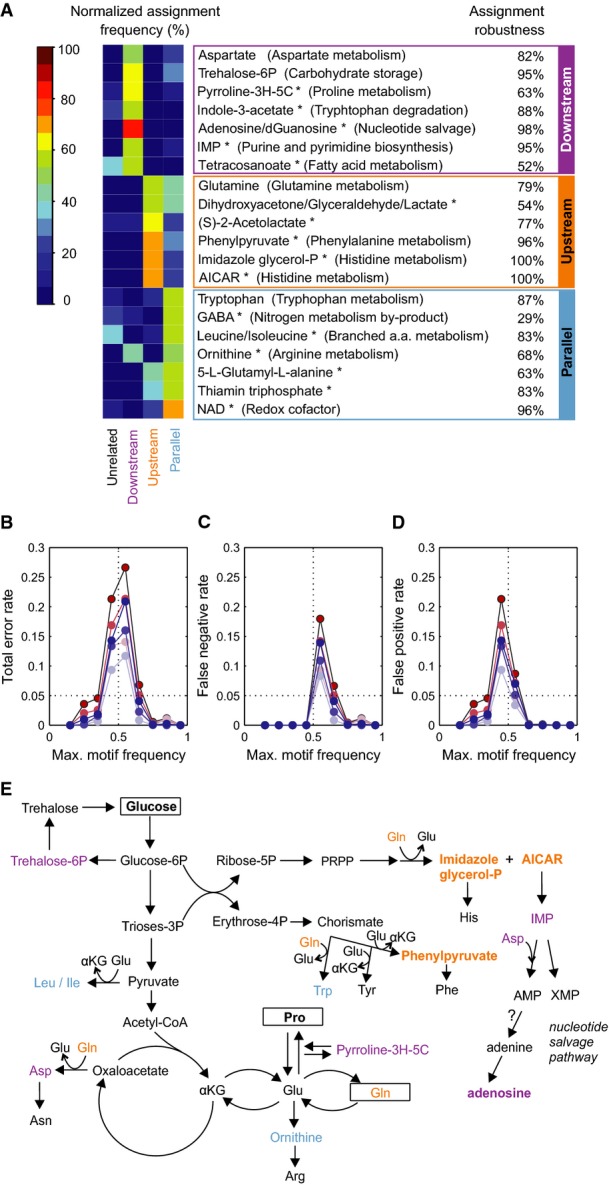
Classification of metabolite functions in nitrogen regulation

While most metabolites were assigned to the “unrelated” network, our method revealed seven “downstream”, seven “parallel”, and six “upstream” metabolites to TORC1 (Fig5A and Supplementary Table S9). To analyze the robustness of the assignments and how motif assignments depend on the prior biological knowledge on TORC1-controlled TFs and their controlled genes, we considered two scenarios: (i) prior knowledge is correct but limited—we know fewer than the 11 reported TFs directly controlled by TORC1—and (ii) knowledge on TORC1-controlled TFs is incomplete, meaning that additional TFs could be directly controlled by TORC1. We investigated both scenarios for different numbers of TFs affected, and we refer to them as “−3 … −1 TFs” and “+ 1 … + 3 TFs” scenarios, respectively (see Materials and Methods for details). Computing motif assignments for all metabolites and all perturbation scenarios revealed that the total error rate, defined as the average rate of assignments that differ from the unperturbed analysis, generally is below 30% (Fig5B and Supplementary Table S9). Sufficient prior knowledge on real TORC1-TFs is important (red curves in Fig5B), whereas additional unknown TFs have less effect (blue curves). If we expect errors in assigning motifs by exceeding the 50% motif frequency threshold, these errors will be false negatives (no motif is assigned although it should be, Fig5C), rather than false positives (assignment of spurious motifs, Fig5D). In particular, all assignment frequencies that exceed approximately 70% have practically zero false-positive and false-negative rates on average. For our original motif assignments shown in Fig5A, we find generally high motif robustness as quantified by the motif recovery stability for the + 3 TFs case. This scenario gives a lower bound in our application-relevant +TF scenarios; previous experimental analyses provide us with high confidence for the validity of the 11 known TORC1-TFs.

Heat-map of the percentage (%) of the normalized assignment frequency of the metabolites over 48 different methods of probabilistic computation. A metabolite is associated with a network if the probability of assignment to this network exceeds 50% for a particular computation method. Metabolites semi-quantified with untargeted FIA-QTOF-MS are marked with *. Assignment robustness scores are the recovery frequencies of the network associations assuming that three additional, random TFs are directly controlled by TORC1 (*N* = 100 samples). Pyrroline-3H-5C: L-1-pyrroline-3-hydroxy-5-carboxylate; Tetracosanoate: tetracosanoate (n-C24:0); dGuanosine: deoxyguanosine; GABA: 4-aminobutanoate; NAD: nicotinamide adenine dinucleotide; αKG: alpha-ketoglutarate; PRPP: 5-phosphoribosyl 1-pyrophosphate. See also Supplementary Table S9.

Robustness of motif assignments in terms of average total error rate for all metabolites binned by their maximal motif assignment frequencies. Red colors show the scenarios of reduced sets of known TORC1-controlled TFs (−3 … −1 TFs, with decreasing color intensity), and blue colors, the scenarios of additional, random TFs assumed to be controlled directly by TORC1 (+3 … +1 TFs, with decreasing color intensity).

Average false-negative motif assignment rates in analogy to (B).

Average false-positive motif assignment rates in analogy to (B).

Mapping of metabolites with normalized assignment frequency over 50% (bold face: > 65%) to central carbon, amino acid, and nucleotide metabolism. Violet, orange, and blue colors correspond to the “downstream”, “upstream”, and “parallel” networks, respectively.

Biologically, many of the metabolites assigned to the “downstream”, “parallel”, and “upstream” motifs are intermediates of amino acid or nucleotide metabolism, processes known to be regulated via TORC1 (Loewith, 2011) (Fig5A and E). The seven “downstream” metabolites include aspartate, T6P, and the purine-related metabolites IMP and adenosine/deoxyguanosine (Fig5A), whose response within the first 10 min suggests post-translational control of an associated enzyme. In the pathway that converts aspartate to threonine, Hom3 and Hom2 catalyze the first two reactions and Thr4 the final conversion to threonine; all three are phospho-enzymes and their deletion causes growth defects with rapamycin (Chan *et al*, 2000). Continuous accumulation of aspartate over at least 20 min (Fig3A), but not of threonine or any of the four pathway intermediates upon TORC1 inactivation, therefore suggests regulation of Hom3 and/or Hom2 activity by TORC1-dependent (de)phosphorylation. T6P is a precursor for the reserve carbohydrate and stress protectant trehalose. Two enzymes of the T6P-synthetase/phosphatase complex that catalyzes T6P production/degradation, Tsl1 and Tps3, were previously found to be hyperphosphorylated in rapamycin-treated yeast (Soulard *et al*, 2010). T6P association with the downstream motif may therefore be a consequence of TORC1 phospho-regulation of the T6P-synthetase/phosphatase complex. The purine-related metabolites are intermediates of DNA and RNA degradation and nucleotide salvage pathways (Fig5E). Deamination of AMP to IMP, catalyzed by the tetrameric Amd1 that becomes dephosphorylated upon rapamycin treatment (Soulard *et al*, 2010; Loewith, 2011), is important for rapid and energy–cost-efficient adaptation of nucleotide pools during changing conditions (Walther *et al*, 2010). Increasing IMP concentration with TORC1 activation during the upshift and the opposite response upon rapamycin treatment (Fig3B) suggest that TORC1 activates Amd1 activity, possibly to avoid accumulation of the allosteric effector AMP, in addition to the well-known TORC1-dependent transcriptional regulation of purine metabolism (Loewith, 2011). “Parallel” motif metabolites are predicted to affect transcription in a TORC1-independent manner (Fig5A), and transcriptional induction of aromatic amino acid catabolism genes by tryptophan has already been described (Iraqui *et al*, 1999).

The six “upstream” metabolites represent candidate endogenous signals to modulate TORC1 activity. Our analysis quantitatively supports the previously raised hypothesis of glutamine being a TORC1 signal in yeast (Crespo *et al*, 2002; Gaubitz & Loewith, 2012). Leucine, in contrast, is assigned to the “parallel” network, which renders its hypothetical role of a natural TORC1 input (Gaubitz & Loewith, 2012) unlikely. Strikingly, the “upstream” network associated glutamine, AICAR, and imidazole glycerol-P, all of which participate in a single reaction at the branch point between histidine and purine nucleotide metabolism (Fig5E). In particular, AICAR is a promising candidate to coordinate amino acid and nucleotide metabolism in response to nitrogen availability because: (i) it stimulates *in vivo* interactions between the phosphate metabolism transcription factor Pho2 with either Pho4 or the regulator of purine nucleotide and histidine metabolism Bas1 (Pinson *et al*, 2009; Ljungdahl & Daignan-Fornier, 2012), and (ii) TORC1 responds (weakly) to histidine deprivation (Binda *et al*, 2009).

## Discussion

Metabolites are top signal candidates to drive rapid cellular adaptations to environmental change because they are amongst the first responding cellular constituents. However, their transient and weak interactions with proteins, potentially leading to immediate allosteric regulation, are notoriously difficult to assess (Gerosa & Sauer, 2011; Link *et al*, 2013). Hence, the field is replete with speculation on the actual signals that govern kinase activity, and TORC1 is no exception (Loewith & Hall, 2011). Here, we developed a generic experimental/computational framework to infer causal relationships within intertwined regulatory networks that combines a probabilistic, model-based method with dynamic metabolite and transcript data. The key novelty of the approach is to correlate the experimentally observed dynamics via prototype models that integrate prior network information systematically. This enables predictions of metabolites that best explain the dynamics of the global transcriptional responses by acting (directly or indirectly) upstream, downstream, or in parallel to a signaling pathway of interest.

Beyond confirming the hypothesized TORC1 signal glutamine (Crespo *et al*, 2002; Gaubitz & Loewith, 2012; Stracka *et al*, 2014), we identified other, putative endogenous inputs into the upstream network of TORC1 signaling. Given our prediction that AICAR acts upstream and IMP, the next intermediate in purine synthesis, acts downstream of TORC1, it is tempting to speculate that TORC1 (or an upstream effector) senses AICAR to regulate the activity of the reaction from AICAR to IMP that is catalyzed by the phosphoproteins Ade17 (Holt *et al*, 2009) or its paralog Ade16 (Soufi *et al*, 2009). By predicting tryptophan to regulate its own synthesis in a TORC1-independent manner, we capture also one of the few known direct interactions of metabolites with yeast transcription factors (Iraqui *et al*, 1999). Downstream of TORC1, we found metabolic imprints of several TORC1-dependent post-translational modifications, including the previously hypothesized T6P-synthase/phosphatase (Soulard *et al*, 2010), whose increased activity accumulates the stress protectant trehalose, the tetrameric Amd1 in purine metabolism (Soulard *et al*, 2010; Loewith, 2011), whose activation is important for efficient adaptation of nucleotide pools during changing conditions (Walther *et al*, 2010), and the novel TORC1-dependent regulation of threonine biosynthesis at Hom3 and/or Hom2. Through a combination of the here reported metabolomics data with phosphoproteomics data, TORC1-dependent activation of T6P-synthase and Amd1 as well as inhibition of Hom3 were independently confirmed (Oliveira *et al*, 2015).

In contrast to earlier approaches, our co-design of perturbation experiments and a probabilistic analysis framework integrates heterogeneous dynamic data and exploits existing network knowledge (e.g. TORC1 controlled genes), prototypic interaction classes (network motifs), and approximations of biological control mechanisms (dynamic dependence). We expect that our approach for model-based data integration will extend to other (nutrient) signaling networks because, while the formalization of the prior knowledge is application specific, the approach itself is generic: target sets, network motifs, and temporal relations of events and variables could be formulated analogously for other applications. Note, however, that the approach may not be applicable to existing datasets in retrospect: a co-design of experiments is critical, for example, because equivalent perturbations of signaling via natural inputs and drugs (here: amino acids and rapamycin, respectively) are needed for the inference approach. Another possible limitation concerns the availability of prior knowledge on, for example, transcriptional regulation. Here, we employed only experimentally documented interactions between TORC1-controlled transcription factors and target genes, but already general sampling biases in the well-characterized yeast transcriptional network could lead to biased inference results. However, our robustness analysis indicates that such confounding factors have less severe effects on prediction accuracy than limited biological knowledge. In addition, the approach requires a careful definition of the “prototypic” motifs (see also Supplementary Text S1), but this cannot guarantee that all possible causal interactions will be identified (e.g. “hybrid” motifs in which a metabolite has multiple roles in the network may be missed).

Future extensions of the computational method to counteract these limitations could incorporate mechanistic details, prior information such as transcriptional or metabolic network structures, and quantitative features of transcriptional control (using, correspondingly, more detailed models) to improve resolution and confidence of inferred causal relationships. For example, the structure of a metabolic network could provide constraints because any change in a metabolite concentration should be preceded by changes in educt concentration(s) in the corresponding reaction(s). In terms of experimental data, constant protein abundances allowed us to focus on metabolite and transcript dynamics. With more detailed dynamic measurements, such as (phospho-)proteomics data, it should become possible to unravel regulatory interactions across multiple network types and timescales, addressing one of the most prominent current challenges for data integration in complex cellular networks. Provided that the topology of transcriptional or phosphorylation regulation networks, or substantial parts thereof, is known, the presented approach is also applicable to regulation in healthy and diseased higher cells.

## Materials and Methods

### Strain and growth conditions

Throughout we used the prototrophic *S. cerevisiae* strain YSBN6 (Canelas *et al*, 2010). Precultures were prepared in 500-ml shake flasks with 50 ml yeast minimal medium, grown for 24 h at 30°C and 300 rpm. Bioreactor batch cultures of 2.5 l medium in a 3.1-l KLF glass vessel (Bioengineering AG, Wald, Switzerland) were inoculated to a starting optical density at 600 nm (OD_600_) of 0.03–0.08, and grown at 30°C, pH 5, impeller speed of 1,000 rpm, and sparging with 3 l air per min. Growth was monitored by OD_600_ measurements and the concentration of CO_2_ in the reactor exhaust gas stream. The yeast minimal medium (YMM) contained (per liter): 20 g d-glucose, 5 g K_2_SO_4_, 3 g KH_2_PO_4_, 0.5 g MgSO_4_·7H_2_O, 15 mg EDTA, 4.5 mg ZnSO_4_·7H_2_O, 0.3 mg CoCl_2_·6H_2_O, 1.0 mg MnCl_2_·4H_2_O, 0.3 mg CuSO_4_·5H_2_O, 4.5 mg CaCl_2_·2H_2_O, 3.0 mg FeSO_4_·7H_2_O, 0.4 mg NaMoO_4_·2H_2_O, 1.0 mg H_3_BO_3_, 0.1 mg KI, 0.05 mg biotin, 1.0 mg Ca-pantothenate, 1.0 mg nicotinic acid, 25 mg inositol, 1.0 mg pyridoxine, 0.2 mg p-aminobenzoic acid, 1.0 mg thiamin, and 10 mM potassium hydrogen phthalate buffer (pH 5). Nitrogen source supplementation was as followed (final concentrations): “preculture-YMM” with 1 g/l of either l-glutamine or l-proline (for cultures initially growing in glutamine or proline, respectively); “downshift-YMM” with 500 mg/l of l-glutamine and 250 mg/l of l-proline; “rapamycin-YMM” with 1 g/l of l-glutamine; and “proline-YMM” with 200 mg/l of l-proline. All experiments were performed in three independent biological replicates.

### Dynamic perturbations and sampling times

#### Proline to glutamine upshift

The initial culture was inoculated in “proline-YMM”. At OD_600_ = 1.07 ± 0.24 (*N *=* *3, SD), still in exponential growth, glutamine was instantaneously added into the bioreactor to a final concentration of 400 mg/l. The time of glutamine addition is referred to as the “time of shift”.

#### Glutamine to proline downshift

The initial culture was inoculated in “downshift-YMM”, containing both glutamine and proline as nitrogen sources. At appropriate intervals, the concentration of glutamine in the broth was monitored by using at-line direct flow injection MS analysis on an Agilent LC-QTOF system, allowing readouts within < 2 min after sampling. The time at which glutamine was not detected anymore by direct flow injection MS is referred to as the “time of shift”, at an OD_600_ of 0.84 ± 0.02 (*N *=* *3, SD).

#### Rapamycin pulse

The initial culture was inoculated in “rapamycin-YMM”. At OD_600_ = 0.85 ± 0.08 (*N *=* *3, SD), still in exponential growth, rapamycin was instantaneously added into the bioreactor to a final concentration of 400 μg/l. The time of rapamycin addition is referred to as the “time of shift”.

#### Sampling times and sampling setup

Throughout the cultivation, we monitored OD_600_ and extracellular concentrations of glucose, glutamine, proline, ethanol, glycerol, acetate, and pyruvate (Supplementary Table S2). Immediately before and for 120 min after the shift, samples for determination of mRNA levels, protein abundances, intracellular metabolites, cell physiology, cell size and DNA content were taken as shown in Fig1B. Steady-state samples taken a few minutes before the shift are also referred to as sample time 0 min (*t*_0_), since concentrations at this time scale can be assumed as constant. The high sampling frequency following the shift was enabled by independent sampling ports, installed at the bottom of the reactor, specific for each measurement type. Sampling for intracellular metabolomics was performed by an automated sampling device with a vacuum pump to maintain a rapid and reliable sampling frequency.

### Determination of biomass and extracellular metabolite concentrations

Biomass concentration was determined as OD_600_ in a spectrophotometer. The correspondence to dry cell weight (DCW) for the YSBN6 strain growing in minimal media was initially determined to be 0.463 gDCW/l/OD_600_. Extracellular concentrations of glucose, ethanol, acetate, glycerol, succinate, and pyruvate were determined with an HPLC system (Agilent HP1100), equipped with a polymer column (Aminex HPX-87H from Bio-Rad) (Buescher *et al*, 2012). Extracellular concentrations of glutamine and proline were determined by GC-MS. Sample derivatization and GC-MS operation were adapted from Zamboni *et al* (2009) with norvaline and glutarate as internal standards. In all cases, quantification was achieved with pure chemicals obtained from Sigma as external standards.

### Determination of intracellular metabolite levels

#### Quenching and extraction

Culture broth was quickly quenched into a tube containing (1:4 v/v) 60% (v/v) methanol buffered with 10 mM ammonium acetate (pH 7.5), precooled at −40°C, and pelleted by centrifugation. Intracellular metabolites were extracted from the pellet with 1 ml 75% (v/v) ethanol buffered with 10 mM ammonium acetate pH 7.5, at 80°C for 3 min. Samples for LC-MS/MS were supplemented with 50 μl ^13^C-labeled internal standard before extraction. After centrifugation for 5 min at 3,500 *g* at −9°C, the extracts (supernatant) were dried completely in a vacuum centrifuge (Christ-RVC 2–33 CD plus, Kuehner AG, Birsfelden, Switzerland). The extracts were resuspended in water before analysis.

#### LC-MS/MS (targeted metabolomics)

Liquid chromatography coupled with mass spectrometry was adopted from Buescher *et al* (2010), including the selected reaction monitoring (SRM) transitions used (Supplementary Table S7). Specifically, liquid chromatography separation was achieved by an ion pairing-reverse phase method implemented on a Waters Acquity UPLC (Waters Corporation, Milford, MA, USA) using a Waters Acquity T3 end-capped reverse phase column with dimensions 150 × 2.1 mm × 1.8 μm (Waters Corporation). The metabolites were ionized with a heated electrospray ionization source and detected with a Thermo TSQ Quantum Ultra QQQ mass spectrometer (Thermo Fisher Scientific, Waltham, MA, USA) using a heated electrospray ionization source. The MS was operated in negative mode with SRM. Data acquisition and peak integration were performed with the Xcalibur software version 2.07 SP1 (Thermo Fisher Scientific) and in-house integration software. Peak areas were normalized to fully ^13^C-labeled internal standards and absolute quantification of metabolites was achieved with linear calibration curves of standards. Finally, concentrations were normalized to the amount of biomass.

#### FIA-QTOF-MS (untargeted metabolomics)

Sample extracts were analyzed by flow injection into a time-of-flight MS (6,550 Series QTOF, Agilent Technologies) operated in the negative ionization mode. High-precision mass spectra were recorded from 50 to 1,000 *m/z* and analyzed as described previously (Fuhrer *et al*, 2011). Each sample was injected and measured twice. Ions were annotated to yeast metabolites based on accurate mass with tolerance of 0.001 Da. Only high confidence annotations were used, for a total of 186 unique *m/z* ions annotated to 256 yeast metabolites (Supplementary Table S7). Relative intensities were normalized to the amount of biomass. The overall performance of the untargeted method was compared against the LC-MS/MS measurements for the common metabolites measured in both platforms (Supplementary Fig S6), and generally the Spearman correlation coefficient was high for those metabolites changing by two-fold or more.

### Determination of mRNA levels

To cover the entire dynamics for transcriptome analysis, 14 or 15 samples were processed per nutritional shift from one representative bioreactor experiment (Supplementary Table S5), and samples from time points −10, 7, and 24 min in the triplicate experiments allowed to statistically assess biological variability, yielding a total of 43 array images. Culture broth was quickly quenched into a tube containing (1:1 v/v) methanol precooled at −40°C, and pelleted by centrifugation. Pellets were washed once with 10 ml ice-cold water. Total RNA extraction was done using the RNeasy kit (Quiagen), including on column DNase treatment for 20 min. The manufacturer's instructions were followed except that we used lysing matrix C for yeast RNA extraction from MP Biomedicals (Santa Ana, CA, USA). Quality of the isolated RNA was determined with a NanoDrop ND 1000 (NanoDrop Technologies, Delaware, USA) and a Bioanalyzer 2100 (Agilent, Waldbronn, Germany). Only samples with a 260/280 nm value between 1.8–2.1 and a 28S/18S value within 1.5–2 were further processed. Total RNA samples (50 ng) were reverse-transcribed into double-stranded cDNA and then *in vitro* transcribed in the presence of biotin-labeled nucleotides using GeneChip® 3′ IVT Express Kit (Affymetrix Inc., P/N 901229). The quality and quantity of the biotinylated cRNA were determined using NanoDrop ND 1000 and Bioanalyzer 2100.

Biotin-labeled cRNA samples (15 μg) were fragmented randomly to 35–200 bp at 94°C in fragmentation buffer (Affymetrix Inc., P/N 900371) and were mixed in 300 μl of hybridization mix (Affymetrix Inc., P/N 900720) containing hybridization controls and control oligonucleotide B2 (Affymetrix Inc., P/N 900454). Samples were hybridized to GeneChip® Yeast 2.0 arrays for 16 h at 45°C. Arrays were then washed using an Affymetrix Fluidics Station 450 FS450_0001 protocol. An Affymetrix GeneChip Scanner 3000 was used to measure the fluorescent intensity emitted by the labeled target. The raw CEL files were normalized using R/Bioconductor, Affy package (background correction: RMA; only perfect-match probes; probe intensity normalization: QSPLINE; expression index calculation: Li and Wong method). The resulting dataset with one intensity per ORF per time point per experiment was the final working dataset. For clustering and PCA analysis, all 25 CEL files comprising the three complete time series (eight in N-upshift, eight in rapamycin and nine in N-downshift) were normalized together, allowing the relative comparison of transcripts across all experiments. For statistical dependency analysis, all CEL files from each specific shift, including the replicates, were normalized together (14 in N-upshift, 14 in rapamycin and 15 in N-downshift), allowing adequate statistical treatment for each shift. Raw data are deposited in NCBI GEO under the accession number GSE54852.

### Determination of protein levels

Protein samples were taken by addition of trichloroacetic acid directly to the yeast culture, followed by cell lysis using glass beads beating in condition of 8 M urea. As internal standard, a ^15^N stable-isotope labeled protein reference yeast extract was spiked into all samples. After reduction, alkylation and trypsin digestion peptide mixtures were purified using C18 cartridges, and all protein samples were assayed in biological triplicates.

SRM measurements were performed on a TSQ Vantage QQQ mass spectrometer (Thermo Fischer Scientific) equipped with a nano-electrospray ion source and a nano-LC system (Eksigent). For all targeted proteins, proteotypic peptides and optimal transitions for identification and quantification were selected based on two high-quality SRM assay repositories of *S. cerevisiae*: (i) A proteome-wide mass-spectrometric map generated from synthetic reference peptides (Picotti *et al*, 2013) (accessible at www.srmatlas.org/yeast/) and (ii) a yeast ion-trap consensus spectral library from the National Institute of Standards and Technology (NIST) (built 2009, accessible at http://peptide.nist.gov). Relative protein quantification was carried out based on the ratio between summed transition areas for light (endogenous) and heavy (^15^N reference) peptide form. For proteins quantified with several peptides the average peptide ratio was used to compute the protein ratio. The quantitative SRM dataset has been deposited to the public repository “Panorama” and is accessible via the link https://daily.panoramaweb.org/labkey/project/Aebersold/ludwig/BigY_SRMdata/begin.view.

### Analysis of transcriptome and metabolome data

#### Principal component analysis (PCA)

PCA was performed with the R-package pcaMethods (method ppca) on the entire transcript set (5,716 transcripts), metabolic gene set [909 transcripts encoding for metabolic enzymes described previously (Herrgard *et al*, 2008)] and LC-MS/MS metabolome set (42 metabolites). Transcript and metabolite intensities were mean-centered by subtracting the average intensity of each transcript/metabolite over the entire samples and scaled to unit variance by dividing by the standard deviation.

#### Onset times

Onset times, defined as the time for a transcript to reach half of its maximum intensity change, were determined by fitting a six-parameter impulse model (Chechik & Koller, 2009) to the dynamic transcript profiles. The impulse model fit was implemented in Matlab, and the parameters h0, h1, h2, t1, t2, and beta that best fit the data were identified based on an optimization problem formulated as minimization of unconstrained variables. By interpolation of the dynamic transcript response given by the impulse response, we calculated the onset time *t*_1/2_.

#### Clustering

Clustering was applied to the dataset containing all measured transcripts over the complete time-course datasets for the three shifts (matrix size: 5,716 transcripts × 25 samples). Model selection and statistical validation was performed with the principle of Approximation Set Coding to determine the most predictive clustering and the number of clusters (Chehreghani *et al*, 2012), which determines the best tradeoff between informativeness and stability of the clustering solutions. Here, it has been employed to compare two relational cost models: pairwise clustering (Hofmann & Buhmann, 1997) and correlation clustering (Bansal *et al*, 2004) with Pearson correlation similarity. Pairwise clustering yielded a higher approximation capacity than correlation clustering, that is a larger number of bits-per-sample which are reliably extracted from the data. For each cluster, a representative time trajectory was estimated by the average value of the members of the cluster at each time point.

### Probabilistic assignment to network motifs

#### Interpolation of metabolite and transcript data

Depending on how we computed the “dynamic dependence” (see below), we either used interpolated trajectories of the average (across three biological replicates) response of each metabolite in every shift, or we interpolated each biological replicate independently for every shift. In both cases, we used two interpolation techniques: (i) linear interpolation, and (ii) cubic smoothing spline interpolation. We interpolated the experimental time points between 0 and 10 min of the metabolic response. To estimate the rate of change of each transcript in time (that is, its time derivative), we interpolated each transcriptional time course (experimental time points between 0 and 24 min of the transcriptional response, evaluated in equally spaced intervals of 1 min) with cubic smoothing splines and differentiated the resulting polynomials. All the interpolated curves were sampled every minute to establish sampled datasets.

#### Computation of “dynamic dependence”

To compute the dynamic dependence between every metabolite–transcript pair for each experiment, we employed the sampled version of distance correlation (Székely *et al*, 2007). The computation of a distance correlation between a set of pairwise samples (within a 10-min interval) of a metabolite–transcript pair resulted in a dynamic correlation value of that pair for that particular time interval. Since the experimental measurements are noisy, we also added Gaussian noise to the pairwise samples (with coefficient of variation equal to 0.1. 0.2, 0.3, and 0.4). Causality was introduced by computing the dynamic correlation between each metabolic trajectory and the derivative of each transcriptional response, and by making use of ten “delayed” versions of the transcriptional response for the computation of the distance correlation (a 10-min window of the initial transcript–trajectory was used in every case, which was moved by 1 min until the 20^th^ min of the transcriptional response). Hence, for each pair of metabolite–transcript, we computed 11 values (each assigned to one transcriptional “delay” value) of dynamic dependence for each experimental case. To achieve robust results, we used three definitions of pairwise correlations: (i) average trajectories: average metabolite samples (over biological replicates) and transcript samples in time define two one-dimensional feature vectors; (ii) individual trajectories, variant 1: the one-dimensional vector of metabolite responses is constructed from all biological replicates; and (iii) individual trajectories, variant 2: the metabolite and transcript vectors are both three-dimensional (for the replicates). With these three variants, two versions of the interpolated metabolite data, and four different levels of variance for the Gaussian noise, we computed the dynamic dependence for each metabolite–transcript pair by 24 different methods. Finally, we selected the 10% of the transcripts with the highest maximum distance correlation value (across all the transcriptional delays) to define a set of genes associated with a metabolite (significant set of genes of a metabolite; SSG of a metabolite). The “dynamic dependence” feature value of a metabolite used for network assignment was defined as the mean value of the distance correlation values of the SSG of this metabolite.

#### Computation of “representation of TOR genes”

For each metabolite, the feature of “representation of TOR genes” is defined as the normalized fraction of genes in SSG that are known to be directly associated with at least one of the transcription factors Gcn4, Rtg1, Rtg3, Gln3, Sfp1, Fhl1, Ifh1, Msn2, Msn4, Gis1, and Sko1 that are known direct targets of TORC1. The direct association was inferred from the YEASTRACT database (Teixeira *et al*, 2006) using the direct and documented interactions (downloaded on 10 Nov 2011).

#### Bayesian inference framework

Given the computed values for the features of “dynamic dependence” and of “representation of TOR genes” for each experimental condition, we employed Bayes factors (Kass & Raftery, 1995) to perform the probabilistic assignment of each metabolite to the four network motifs. The Bayes factor *B*_*ij*_ is defined as the fraction of the posterior probability of the motif *M*_*i*_ given the data D with the posterior probability of the motif *M*_*j*_ given the data:

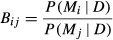
The posterior probability of each motif *M* given the data can be computed via Bayes’ rule as:

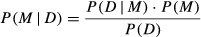
where *P*(*D|M*) is a likelihood that represents the probability that the data are produced under the assumption of the motif *M*, *P*(*M*) is the prior value of the motif *M* (representing the initial degree of belief in the motif), and *P*(*D*) is the evidence of the data. We assumed uniform priors for each metabolite-network assignment such that the Bayes factor *B*_*ij*_ is given by:

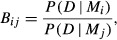
namely the fraction of the likelihoods of the two motifs. The likelihood of assigning a metabolite to a network motif was computed based on the corresponding “motif prototype” values (see Fig4B) and the values of the metabolite-related features compared to the feature distribution of a random set of metabolites (of an initial constant value plus Gaussian noise of same coefficient of variation as in the experimental dataset). Specifically, the likelihood value was defined as the product of the distances of the features’ values on the empirical cumulative density function (eCDF) of the random metabolite set from the “motif prototype” values. When a “motif prototype” value is “R”, then the eCDF distance (of a feature with value **x**) from the median of the random set distribution is computed only when eCDF(**x**) > 0.5. If eCDF(**x**) < 0.5, then the eCDF distance equals zero. Note that we employed two ways to compute the likelihood of assigning a metabolite to a network motif *M*: (i) by accounting for the likelihood in all three conditions, and (ii) by accounting for the likelihood values of the rapamycin downshift and of the nutrient shift with the highest likelihood value. Finally, by computing the values of:

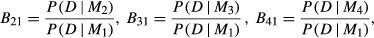
and accounting for the fact that the sum of all four posterior probabilities has to equal one, we can easily compute posterior probabilities of the assignments of each metabolite to the four network motifs.

#### Frequency of assignment to network motifs

Altogether, we performed 48 different probabilistic assignments of each metabolite to the four network motifs (24 variants for computing the dynamic dependence and two variants of determining probabilities of network assignments). For a metabolite, the frequency of assignment to a network motif is defined as the fraction of the methods that support the metabolite-network assignment with more than 50% probability. Note here that although the probabilities of the network assignments always sum up to one, this does not apply to the frequencies of assignments. When, for a metabolite, the assignment probabilities (computed with a particular method) are identical for the four network motifs (i.e. 25%), then this method will increase the frequency of “no network motif”.

#### Robustness of assignments to network motifs

The only prior knowledge we use for the analysis is the set of transcription factors (TFs) that, according to the literature, are direct targets of TORC1 (11 TORC1-TFs). This set of TFs influences directly the values of the feature “representation of TOR genes”, which in turn affects the motif frequency value (fraction of computational methods assigning a metabolite to a specific motif), and hence the final assignment of a metabolite to a network motif via the dominant motif frequency. We performed two types of perturbations to the TORC1-TFs set: (i) We removed all possible combinations of one (−1), two (−2), or three (−3) TFs and recomputed the assignments of the metabolites to network motifs for all possible TF subsets (11, 55, and 155 sets, respectively). (ii) We added one (+1), two (+2), or three (+3) TFs to the TORC1-TFs set by random selection from the yeast TFs not known to be targets of TORC1, with *N = *100 samples each. We call “assignment robustness” the fraction of perturbation sets in which a metabolite is assigned to the same network motif as in the unperturbed case. Supplementary Table S9 contains the “assignment robustness” and the standard deviation of the dominant frequency for every metabolite and for all perturbations cases.

### Data availability

The raw gene expression data comprising all three shift experiments are deposited in the NCBI Gene Expression Omnibus (GEO) under the accession number GSE54852 (http://www.ncbi.nlm.nih.gov/geo/query/acc.cgi?acc=GSE54852). Each individual shift experiment can alternatively be retrieved under accession numbers GSE54844 (N-upshift), GSE54850 (N-downshift), and GSE54851 (rapamycin-induced downshift). The protein quantitative SRM dataset is deposited in the public repository Panorama and accessible via the link https://daily.panoramaweb.org/labkey/project/Aebersold/ludwig/BigY_SRMdata/begin.view. All data were processed as described in Materials and Methods. The Matlab code for the network inference method is provided as Supplementary File S1.

## Supporting Information

Supplementary Text S1

Supplementary File S1. Code and documentation for the network inference algorithm (Matlab)

Review Process File
