# Supplementary material for: Inferring causal metabolic signals that regulate the dynamic TORC1-dependent transcriptome
Source: Mol Syst Biol. 2015 Apr 17;11(4):802. doi: 10.15252/msb.20145475 (PMC4422559; doi:10.15252/msb.20145475)
Supplement: Supplementary file 1 [file msb0011-0802-sd1.pdf]

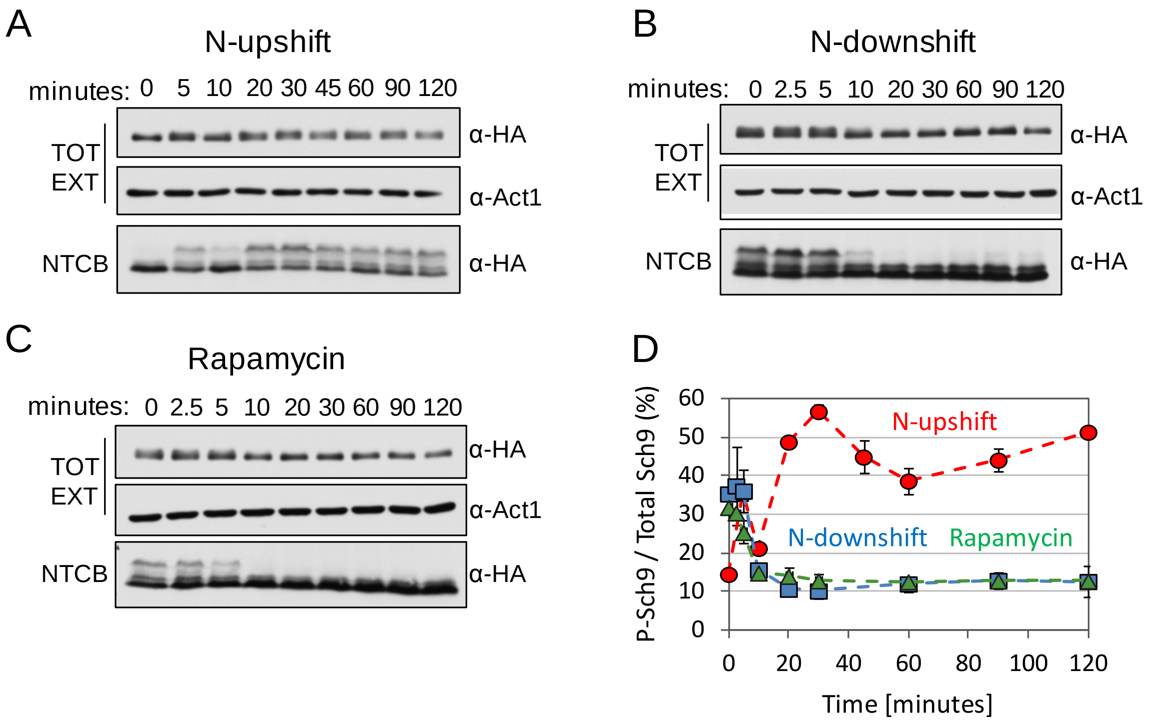

Supplementary Figure 1. Phosphorylated Sch9 as proxy for TORC1 activity following nutrient up- and downshifts and rapamycin treatment. The percentage of phosphorylated Sch9 C-terminus was used as a readout of TORC1 activity, as described in **Supplementary Table 3**, with higher Sch9 phosphorylation corresponding to higher TORC1 activity. **A)** Nutrient upshift. **B)** Nutrient downshift. **C)** Rapamycin treatment. **D)** Quantification of Sch9 C-terminus phosphorylation during nutrient shift and rapamycin treatment (relative to panels A, B and C). Values represent the mean of Sch9 phosphorylation percentage ± s.d. of three biological independent experiments.
