# Supplementary material for: Inferring causal metabolic signals that regulate the dynamic TORC1-dependent transcriptome
Source: Mol Syst Biol. 2015 Apr 17;11(4):802. doi: 10.15252/msb.20145475 (PMC4422559; doi:10.15252/msb.20145475)
Supplement: Supplementary file 2 [file msb0011-0802-sd2.pdf]

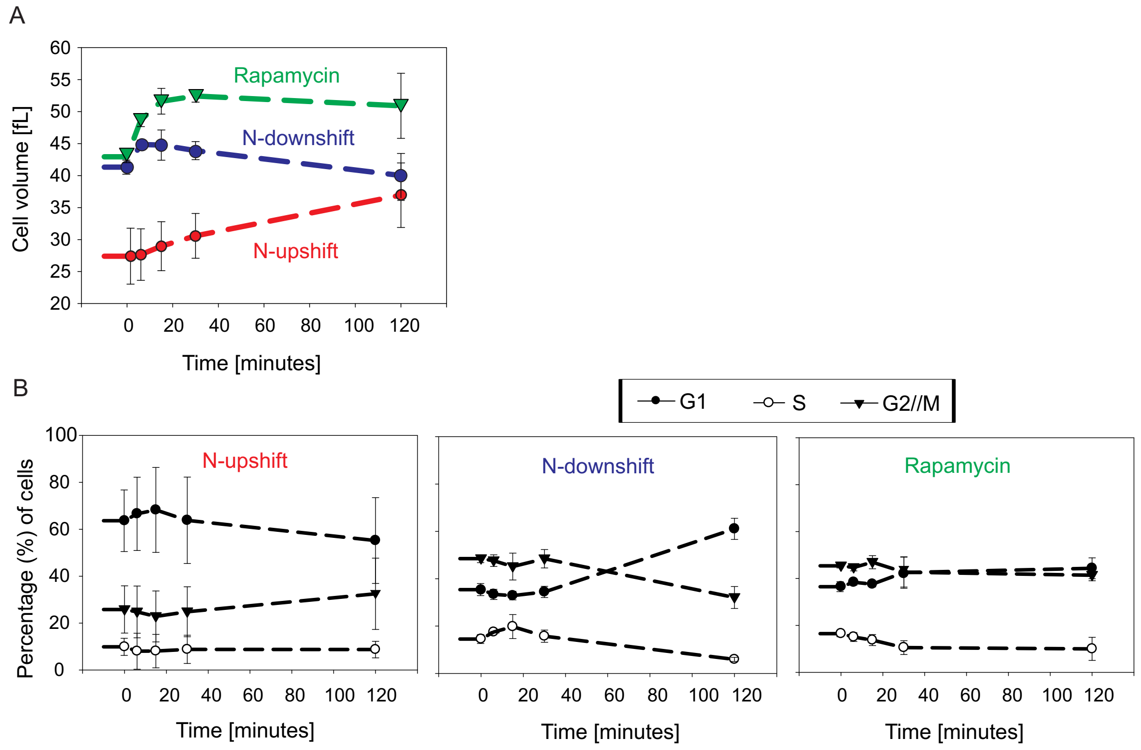

Supplementary Figure 2. Cell volume and DNA content in the three shifts. Samples were taken at steady-state (around 10 minutes before the shift), and after 6, 15, 30 and 120 minutes of the shift, and every point was sampled in three independent biological triplicates (*N*=3) and measured for **(A)** cell volume and **(B)** DNA content as described in **Supplementary Table 4**. Values represent the mean ± s.d. of three biological independent experiments.
