# Supplementary material for: Inferring causal metabolic signals that regulate the dynamic TORC1-dependent transcriptome
Source: Mol Syst Biol. 2015 Apr 17;11(4):802. doi: 10.15252/msb.20145475 (PMC4422559; doi:10.15252/msb.20145475)
Supplement: Supplementary file 3 [file msb0011-0802-sd3.pdf]

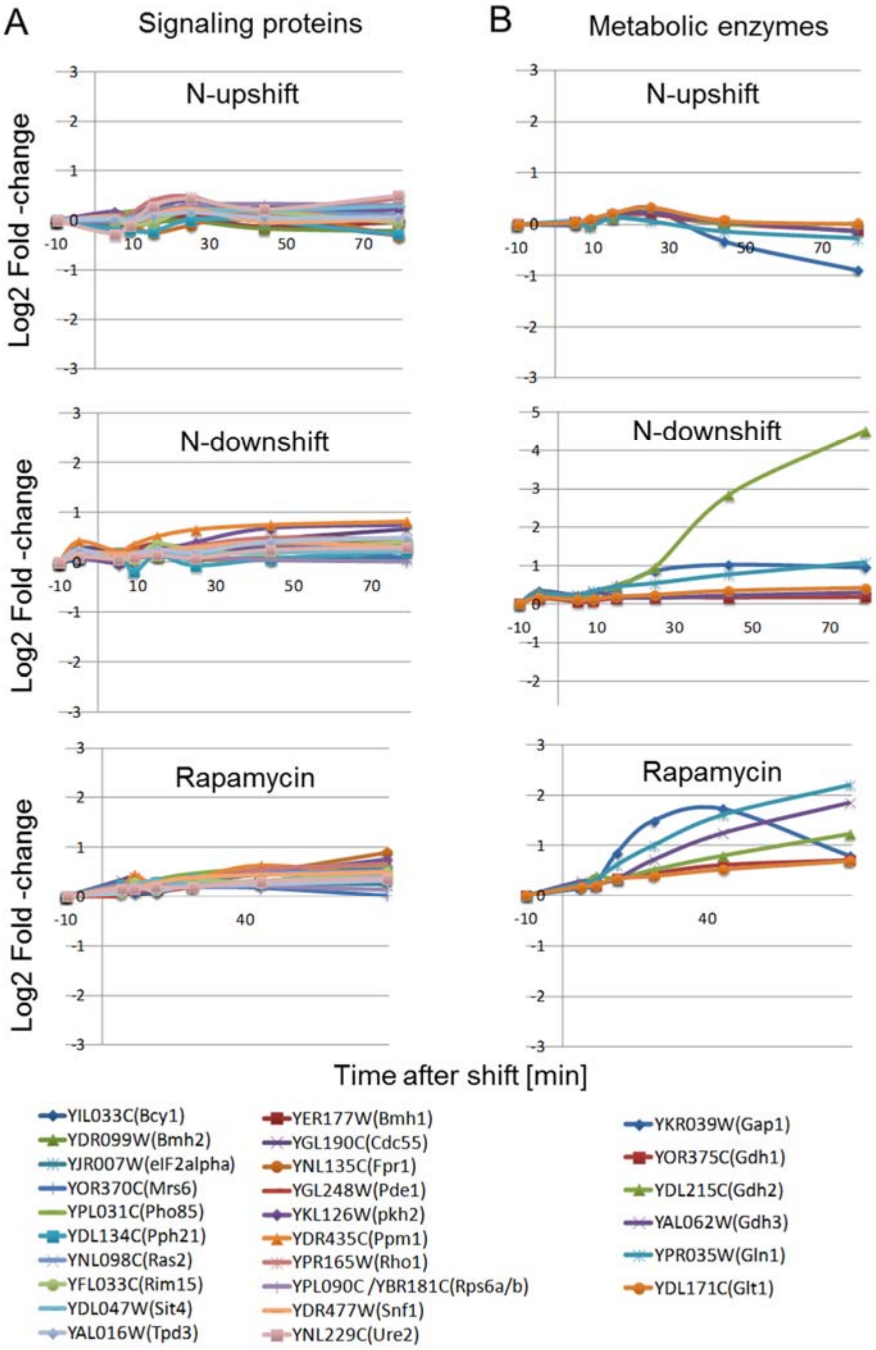

Supplementary Figure 3. Dynamic protein abundance changes over the three shift experiments. Using Selected Reaction Monitoring protein abundance changes for **(A)** 20 targeted signaling proteins and **(B)** 6 targeted enzymes or membrane transporters were monitored over the three shift experiments. The given protein ratios (ratio between the endogenous-light versus reference-^{15}N-labeled protein intensity) were averaged over three biological replicates and normalized to the steady-state time point "-10 minutes" in each shift, respectively. The not averaged and normalized data is given in **Supplementary Table 6**. The complete SRM data set is publicly accessible via the link https://daily.panoramaweb.org/labkey/project/Aebersold/ludwig/BigY_SRMdata/begin.view?
