# Supplementary material for: Inferring causal metabolic signals that regulate the dynamic TORC1-dependent transcriptome
Source: Mol Syst Biol. 2015 Apr 17;11(4):802. doi: 10.15252/msb.20145475 (PMC4422559; doi:10.15252/msb.20145475)
Supplement: Supplementary file 4 [file msb0011-0802-sd4.pdf]

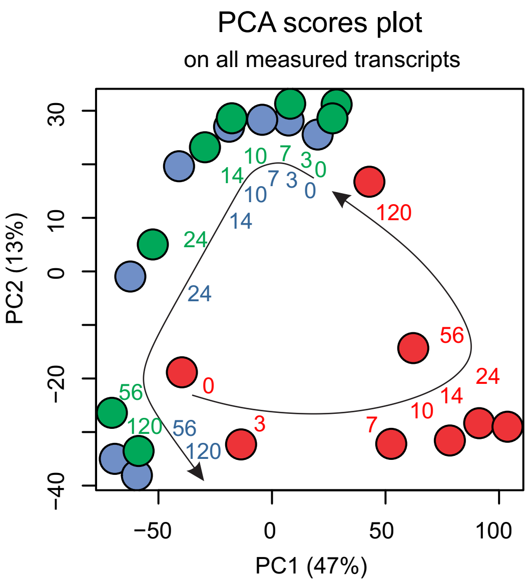

Supplementary Figure 4. PCA on all measured transcripts. Scores plot of the first two principal components (PC1, 2) applied to all genes from all time-point samples across the three shift experiments (red: N-upshift; blue: N-downshift; green: rapamycin-induced downshift). In parenthesis is the variance captured by the principal component.
