# Supplementary material for: Inferring causal metabolic signals that regulate the dynamic TORC1-dependent transcriptome
Source: Mol Syst Biol. 2015 Apr 17;11(4):802. doi: 10.15252/msb.20145475 (PMC4422559; doi:10.15252/msb.20145475)
Supplement: Supplementary file 5 [file msb0011-0802-sd5.pdf]

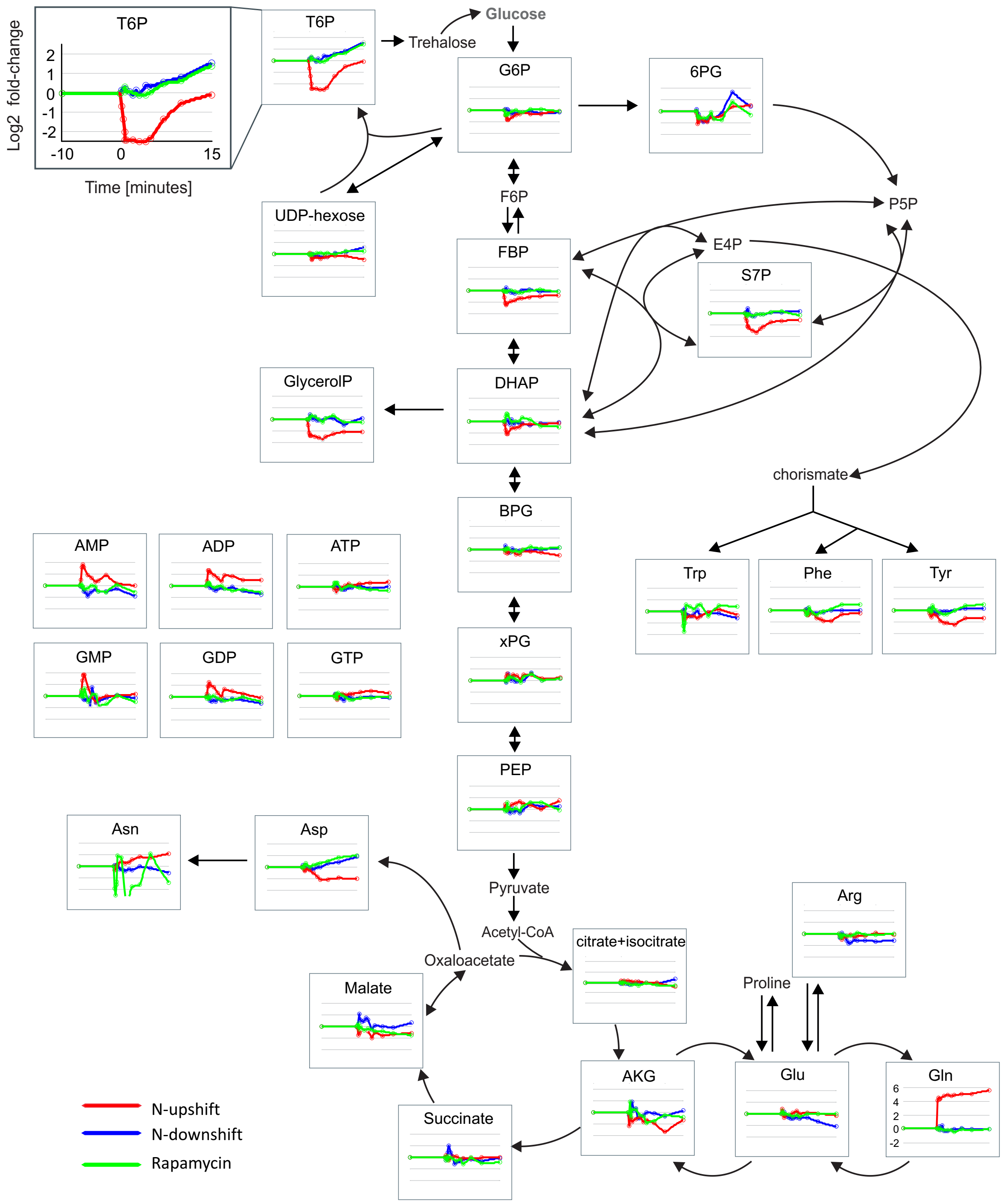

Supplementary Figure 5. Early metabolite changes within central carbon metabolic pathways. Profiles of metabolite changes (normalized to the steady-state measurement) along the first 15 minutes after the shift. Axis scaled as in the larger example of T6P, except the Y-axis of Gln. Only measurements with targeted metabolomics (LC-MS/MS) are shown. Abbreviations: BPG: 2,3-Disphospho-D-glycerate; xPG: 3-Phospho-D-glycerate/2-Phospho-D-glycerate; 6PG: 6-Phospho-D-gluconate; AKG: alpha-Ketoglutarate; FBP: D-Fructose 1,6-bisphosphate; G6P: D-Glucose 6-phosphate; DHAP: Dihydroxyacetone phosphate; GlycerolP: Glycerol 3-phosphate; Arg: L-Arginine; Asn: L-Asparagine; Asp: L-Aspartate; Glu: L-Glutamate; Gln: L-Glutamine; Phe: L-Phenylalanine; Trp: L-Tryptophan; Tyr: L-Tyrosine; PEP: Phosphoenolpyruvate; S7P: Sedoheptulose 7-phosphate; T6P: Trehalose 6-phosphate.
