# Supplementary material for: Inferring causal metabolic signals that regulate the dynamic TORC1-dependent transcriptome
Source: Mol Syst Biol. 2015 Apr 17;11(4):802. doi: 10.15252/msb.20145475 (PMC4422559; doi:10.15252/msb.20145475)
Supplement: Supplementary file 6 [file msb0011-0802-sd6.pdf]

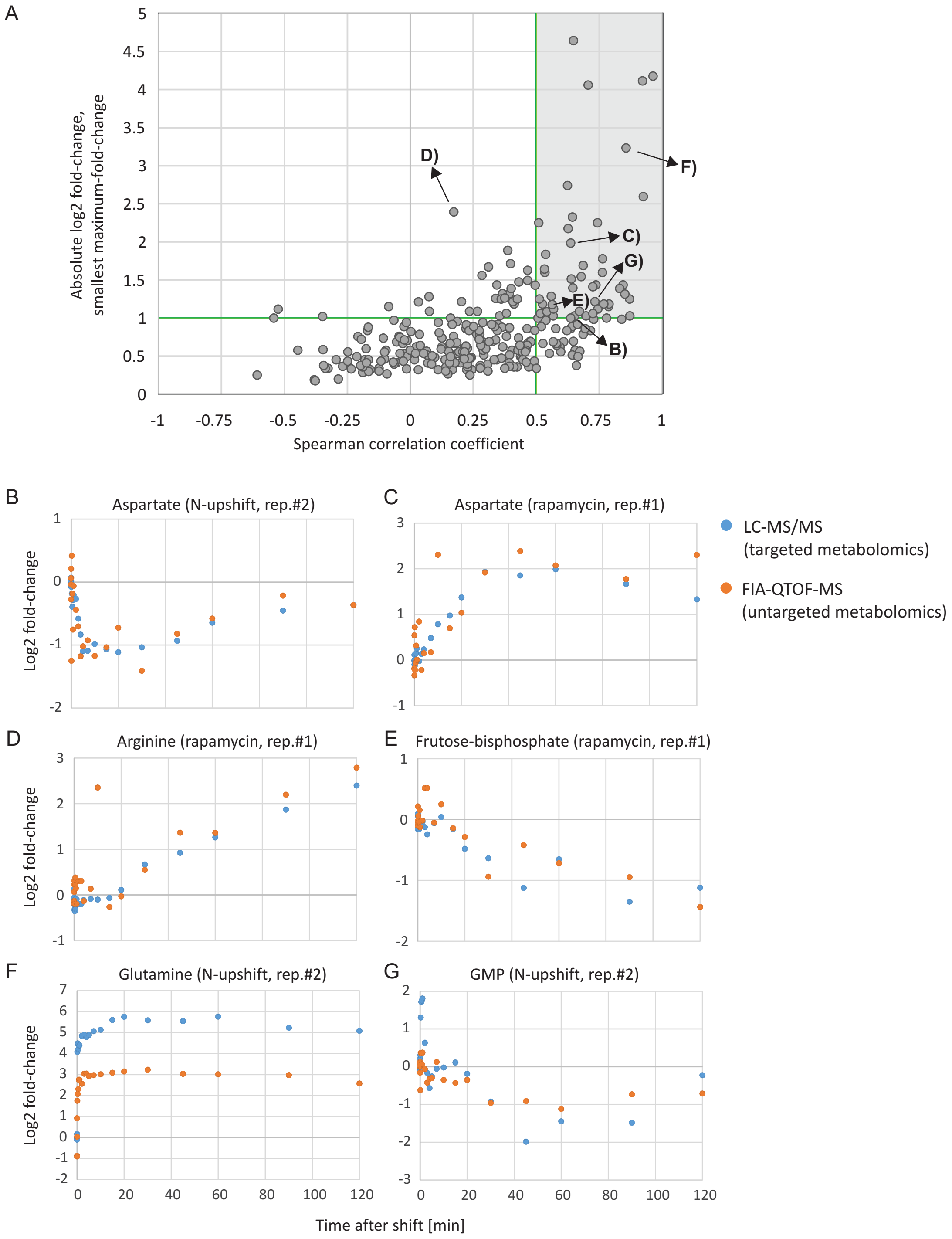

Supplementary Figure 6. Comparative evaluation of the two metabolomic platforms. (A) The Spearman correlation coefficient for 31 metabolites measured both in the LC-MS/MS and FIA-QTOF-MS platforms was calculated for each of the nine experiments performed (three conditions, three replicates each). Correlation coefficients are plotted against the smallest maximum fold-change in either of the platforms. Generally we observed similar profiles in either platforms for changing metabolites. **(B-G)** Example of metabolite profiles changing in both platforms, including glutamine and aspartate identified in our upstream and downstream networks, respectively.
