# Supplementary material for: Inferring causal metabolic signals that regulate the dynamic TORC1-dependent transcriptome
Source: Mol Syst Biol. 2015 Apr 17;11(4):802. doi: 10.15252/msb.20145475 (PMC4422559; doi:10.15252/msb.20145475)
Supplement: Supplementary file 7 [file msb0011-0802-sd7.doc]

**Supplementary Table 1. Experiments nomenclature and times of the shifts relative to the entire fermentation time.** The name of each shift experiment is given in the first column, including in the parenthesis possible alias used throughout the work. Each of the three shift experiments was performed in three independent biological replicate experiments, each labeled with a unique identifier BY00x (original labeling). The time and OD600 at the time of shift for each experiment are indicated in the third and forth column, as well as the precise time of the steady-state sample in the fifth and sixth column. The time of the shift relative to the total fermentation time is henceforth called “time of the shift, t0”.

| **Name of shift experiment** (abbreviations used) | **Experiment label** | **Time of the shift relative to total fermentation time [hours]** | **Biomass con-centration at the time of the shift [OD_600_]** | **Time of the steady-state sample**  **“-10 minutes” relative to the total fermentation time [hours]** | **Actual number of minutes before the shift of the steady-state sample  “-10 minutes” [minutes]** |
| --- | --- | --- | --- | --- | --- |
| **Proline to Glutamine** (N-upshift; Pro->Gln; P2G) | BY003 | 14.75 | 1.02 | 14.58 | -10 |
|  | BY008 | 16.5 | 0.85 | 16.17 | -10 |
|  | BY012 | 15.16 | 1.33 | 14.99 | -10 |
| **Glutamine to Proline**  (N-downshift; Gln->Pro; G2P) | BY009 | 13 | 0.82 | 12.67 | -20 |
|  | BY010 | 11.41 | 0.85^a^ | 11.16 | -15 |
|  | BY013 | 13.1 | 0.86^a^ | 12.83 | -16.2 |
| **Rapamycin-induced downshift** (Rapamycin; Gln->Rap; G2R) | BY005 | 12.08 | 0.76 | 11.91 | -10 |
|  | BY006 | 13.83 | 0.87 | 13.66 | -10 |
|  | BY007 | 12.75 | 0.92 | 12.58 | -10 |

^a^Extrapolated value of OD_600_ based on the annotated time of the shift and on the OD600 measurements during exponential growth, from which an exponential curve fit was obtained.
