# Supplementary material for: Inferring causal metabolic signals that regulate the dynamic TORC1-dependent transcriptome
Source: Mol Syst Biol. 2015 Apr 17;11(4):802. doi: 10.15252/msb.20145475 (PMC4422559; doi:10.15252/msb.20145475)
Supplement: Supplementary file 16 [file msb0011-0802-sd16.pdf]

Supplementary Text 1: Selection and identifiability of network motifs

The logic of the computational approach depends on a set of conditions derived from the prior knowledge on interactions in TORC1 signaling (e.g., the existence of TORC1-controlled transcription factors), on responses to perturbations in external conditions (in particular, similarity of responses to rapamycin and to the glutamine-to-proline shift), and on time-scales of the dynamic responses (e.g., faster metabolic than gene regulatory responses, such that at the investigated time-scales, feedback control of metabolism by gene expression can be excluded). To obtain non-random assignments to the dynamic dependency between metabolites and genes (DD) and to the representation of genes that are targets of transcription factors known to be directly controlled by TORC1 (RG) features (see **Methods** for details on the computation of these features), the following conditions have to be met:

1. Non-random DD in nutrient shifts: A metabolic state is predictive of a change in transcription rate, which implies that a metabolite concentration without (MET→GENES) or with (MET→TORC1→GENES) involvement of TORC1 signaling influences the rate of gene expression.
2. Non-random DD with rapamycin: Since rapamycin directly inhibits TORC1 and it is not known to have direct / short term metabolic effects, for the metabolic state to be predictive of a change in transcription rate, the metabolite concentration has to be controlled by (and therefore correlated with) TORC1 activity.
3. Non-random RG in any condition: All of the following three conditions have to apply:
    a. A non-random dynamic dependency needs to exist because the RG is computed based on gene sets selected by dynamic dependency; otherwise, an over-representation of TORC1-controlled genes would be possible in a random gene set.
    b. A direct route from TORC1 to a specific gene set exists via TORC1-controlled TFs.
    c. The RG assignment has to be identical in nutrient and rapamycin shifts unless the metabolite is upstream of TORC1.

Focusing on the differences between nutrient- and rapamycin-induced downshifts, 16 definitions of 'prototypic' motifs (the extreme cases in which RD or DD are either random or maximal) are possible (2 conditions, 2 features per condition, and 2 possible outcomes for each feature). The corresponding 16 different outcomes in terms of DD/RG combinations are summarized in the table below, where 'X' stands for any possible assignment of a feature value ('1' or 'R').

Motif	Gln->Pro		Gln->Rap		Rationale
	DD	RG	DD	RG	
Downstream	1	1	1	1	‘Prototypic’ motifs as shown in **Figure 4A**.
Unrelated	R	R	R	R	
Upstream	1	1	R	R	
Parallel	1	R	R	R	
Not feasible	X	X	1	R	These four outcomes are not considered because they either imply (unknown) metabolic targets of rapamycin or that a majority of genes regulated by TORC1-TFs is unknown (contradicting the hypothesis of our analysis and the fact that the TORC1-controlled transcription network is well-characterized experimentally).
Not feasible	X	X	R	1	These eight outcomes are not feasible because they admit an over-representation of TORC1 targets in a random gene set (note that only seven outcomes are unique, and that one outcome is identical with the category above).
Not feasible	R	1	X	X	
Not feasible	X	R	1	1	These two outcomes are not considered because the route via known TORC1-TFs is not involved during nutrient shifts (see below for details).

Note first that a motif is structurally identifiable when the 6 parameters used for its definition (DD and RG for all three conditions) have different values. The four prototypic motifs used in the analysis are structurally identifiable, as shown by their different parameter values above and in **Figure 4B**. However, this does not imply that the wiring diagram of a prototypic motif (as shown in **Figure 4A**) is unique. For example, the downstream motif can potentially involve various wiring patterns with different time sequences; they all have in common that the metabolite is downstream of TORC1 (see **Figure T1A**). If we denote by ‘Dx’ a specific wiring diagram associated with the downstream motif, the motif D3 (TORC1 controls MET, TORC1 controls GENES through known TORC1-TFs) and the motif D1 (TOR controls MET, MET controls genes through known TORC1-TFs) cannot be discriminated because both admit the same DD and RG values across the 3 conditions.

Outcomes beyond those associated with our prototypic motifs are not feasible biologically or they conflict with essential hypotheses underlying our study. For example, in D6 (**Figure T1B**), with a potentially intuitive wiring TORC1→MET→GENES, the metabolite is downstream of TORC1, and the metabolite controls gene expression through a set of TORC1-independent TFs, while TORC1 does not control this gene set directly. However, when TORC1 is upstream of the metabolite, their time course responses are statistically correlated because TORC1 controls the metabolite abundance. Hence, the TOR response would also be statistically correlated with the same gene-set response. Such an event for our analysis means that TORC1 exerts a control on the gene set, which can be done only through the set of known TORC1-TFs. This is in contradiction with the D6 wiring diagram. Wirings supporting the

aforementioned analysis are the motifs D8-9 that admit a value for the RG parameter in the interval (R, 1) due to the simultaneous effect of known TORC1-TFs and TORC1-independent TFs on the gene set. Since we are not able to set the RG value for these motifs, we did not consider them for the analysis.

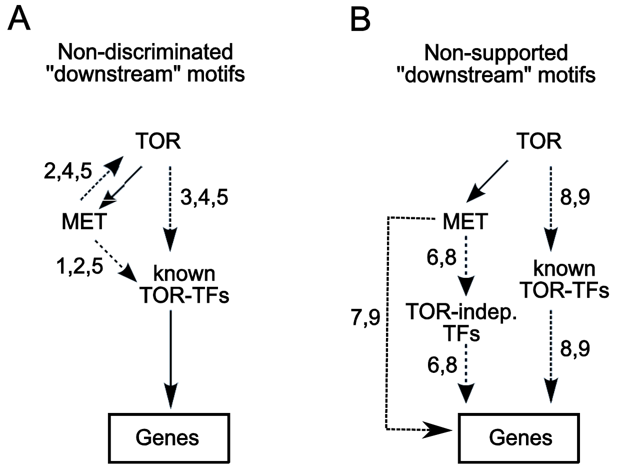

Figure T1: Downstream motifs and network wiring. Nine wiring diagrams associated with the downstream network motifs (D1-9), that cannot be discriminated (**A**), or cannot be supported (**B**) by the analysis. Solid lines exist in all motifs, whereas dashed lines exist only in the motifs that the numbers next to the corresponding arrows designate.
