# Supplementary material for: Inferring causal metabolic signals that regulate the dynamic TORC1-dependent transcriptome
Source: Mol Syst Biol. 2015 Apr 17;11(4):802. doi: 10.15252/msb.20145475 (PMC4422559; doi:10.15252/msb.20145475)
Supplement: Supplementary file 18 [file msb0011-0802-sd18.pdf]

Inferring causal metabolic signals that regulate the dynamic TORC1-dependent transcriptome

Ana Paula Oliveira, Sotiris Dimopoulos, Alberto Giovanni Busetto, Stefan Christen, Reinhard Dechant, Laura Falter, Morteza Haghir Chehreghani, Szymon Jozefczuk, Christina Ludwig, Florian Rudroff, Juliane Caroline Schulz, Asier Gonza lez, Alexandre Soulard, Daniele Stracka, Ruedi Aebersold, Joachim M. Buhmann, Michael N. Hall, Matthias Peter, Uwe Sauer, and Jörg Stelling

Corresponding author: Joerg Stelling, ETH Zurich

Review timeline:

Submission date:	03 June 2014
Editorial Decision:	15 July 2014
Revision received:	20 January 2015
Editorial Decision:	24 February 2015
Revision received:	19 March 2015
Accepted:	23 March 2015

Editor: Maria Polychronidou

Transaction Report:

(Note: With the exception of the correction of typographical or spelling errors that could be a source of ambiguity, letters and reports are not edited. The original formatting of letters and referee reports may not be reflected in this compilation.)

1st Editorial Decision 15 July 2014

Thank you again for submitting your work to Molecular Systems Biology. We have now heard back from the three referees who agreed to evaluate your manuscript. As you will see from the reports below, the referees acknowledge that the presented approach is potentially interesting. However, they raise a series of concerns, which should be carefully addressed in a revision of the manuscript.

Without repeating all the comments listed below, some of the more fundamental issues are the following:
- Follow-up experimentation validating the model's predictions of novel metabolic signals upstream/downstream TORC is required. Reviewer #3 provides constructive suggestions regarding this point.
- Reviewer #1 raises concerns on the choice of motifs and potential issues with motif identifiability.
- The broader applicability of the presented framework should be discussed.

Moreover, the reviewers refer to the need to extend the description of the methodology.

\-

Reviewer #1:

The authors present a novel inference method based on Bayesian modeling to predict the how metabolites regulate gene expression. The method is based on choosing the 'motif' that best explains the dynamics of the global transcriptional responses.

© European Molecular Biology Organization

The method is applied to a new and very interesting by itself data set that combines dynamic (time-series) metabolomic, transcriptomic and proteomic data under three different conditions using wild-type S.cerevisiae cells: two to capture metabolic shifts between poor N-source (proline) and rich N-source (glutamine), and the third to show the effect inhibition of TORC1 by Rapamycin. High changes at the transcriptional level were identified, and to study the possible drivers of these changes via TORC1 the inference model was applied using four different motifs (no-effect, acting upstream, acting downstream, or in parallel with signalling pathways of interest) that were assembled based on prior-knowledge in the literature. The authors conclude from the model and the metabolomics experiments that most of the metabolites were unrelated to the transcriptomics changes, 7 to be downstream of TORC1, 7 parallel and 6 upstream TORC1. They provide partially indirect validation (by testing against literature) and propose new leads for further investigation.

This paper presents a novel approach to an important current problem in the field of systems biology: how to link different cellular processes (metabolism, signalling and gene regulation). As authors state in the introduction, there are only few methods for this, and in particular in the context of dynamics, which is expected to be a key aspect in these processes and their interactions. Hence, methods such as this are highly needed and can have a potential important impact in the community.

The method sounds solid from the mathematical and statistical point of view, and some robustness tests have been done. However, more detail would be useful (as outlined below).
The choice of the case study to illustrate their method is a very adequate one: TOR is a well-known regulator of metabolism, and the knowledge of its role is yeast has been widely study. In addition, yeast is a good model system to perform different 'omics' experiments with expected good quality. Accordingly, the experimental set-up is well defined and the different time-points for the measurements allow to capture the dynamics that are characteristic of nutritional changes at the level of protein and metabolic networks.

While we consider this a potentially important contribution to the field, we feel the following points should be addressed to accurately assess its value.

Major revisions:

The networks motifs were selected based on prior-knowledge, but is not clear why authors have chosen those and then not taken into account other types of motifs such as TOR -> Met -> Genes. Also, the "downstream" network (fig. 4a) is unclear: there is an arrow TOR->genes, and one TOR-> MET, but why not MET-> genes? (as is in e.g. the parallel case)? Even if there is compelling evidence for some motifs over others, it shouldn't be computationally difficult to explore further motifs.

A related point is that of identifiability. Some of the motifs may not be distinguishable from each other - for example, the TOR- > MET- > Genes and the (TOR- > MET & TOR- > Genes) versions may not be distinguishable?

Also what happens if some metabolite has multiple connections in this network, both up- and downstream of TOR?

In general, some more explanation on the choice of motifs and their potential identifiability would be important. Authors could include some analyses to asses this.

Some of the hypothesized candidates were validated with references from the literature. We appreciate that to perform biological validations of the candidates may not be trivial. That said, some experimental validation assessing for instance the post-translational regulation of the downstream candidates of TORC1 would complement the existing validation and provide further evidence of the usefulness of the method. Are any of these possible? We do not consider this a requirement for publication, but certainly would strengthen the paper.

From the (few) existing approaches to link metabolism to regulatory processes, many of them use metabolic (and sometimes regulatory and/or signaling) networks as a basis. In fact, the authors have done important contributions in this area (including recently Oliveira et al. MSB 2012). Can the approach presented here be combined with those methods? At least it should be discussed the

potential relationships.

Although the problem is well contextualised, the methods are not explained in detail, and some aspects are not clear. For instance, it is not clear if the proteomics data-set that was mentioned in the introduction was actually used in the inference method. Or what is the exact nature of the models: in Fig 4B a general ODE equation is described, but what is the exact set of equations (for each of the motifs)?

Minor revisions:

Related to the above point on clarity, some points are present in the methods, but the connection from main text is not clear. For example, it is stated 'For every metabolite [...] and then determined the fraction of genes known to be targeted by TORC1-dependent transcription factors". It is only when one goes to the the materials and methods that is explained how is this done ,the use of the YEASTRACT database, etc - pointers to materials and method would help here.

Authors should make their code available

Authors could elaborate on how this could be applied in other contexts - is this applicable say in human and in particular disease biology?

Suggestions:

A large part of the manuscript is devoted to describe the data (both in text and in figures). We would suggest to shorten this part (and move details to the supplements), and rather explain in more detail the method and the analyses performed with it, as stated above: these are the key novel aspect of this paper, and the harder to grasp, in our opinion.
For example, Figure 2 is used to support the statement that the Rapamycin treatment condition is similar to N-downshift condition (glutamine depletion). Maybe this figure could be part of the supplementary materials and give more emphasis to the methods.

Reviewer #2:

Summary. Cells respond to changing environmental conditions through the integrated use of highly complex signaling, transcriptional and metabolic networks. A major challenge of systems biology is gaining a better understanding of how these networks are interconnected and deconvoluting the sequence of events that results in specific cellular responses. Oliveira and Dimopoulos et al. report construction of a probabilistic framework to integrate dynamic gene expression and metabolite measurements in S. cerevisiae, which they use to infer causal influences that act upstream and downstream of TORC1 in response to alternative nitrogen sources. Their framework integrates time-course data of metabolite, (protein -not compelling) and transcript abundance to determine causal relationships by taking advantage of prior knowledge.
In general, the approach seems sound and the analysis/statistical framework is rigorous, but there are significant concerns with regard to experimentation. Also, for publication in a high profile journal like MSB, the authors need to provide validations for key model predictions.
Major issues
1. While the model predicts several known (or strongly hypothesized) features of TORC1 signaling (e.g., role of glutamine and TORC1-independence of tryptophan synthesis) and the authors provide interesting speculations based on the model's predictions (e.g., TORC1-dependent (de)phosphorylation of Hom3/Hom2 and Tsl1/Tps3, and TORC1 role in AICAR->IMP conversion), it is unclear what has been learned about TORC1 signaling (other than model-derived speculation). To increase biological significance of the work, the authors should provide compelling evidence and validation for other predictions. For publication in a top-tier journal like MSB, such validations would be critical.

2. I presume the proposed framework could be used with the Bacillus subtilis data generated by this group in Buescher et al. 2012. It would be interesting to see how the proposed framework performs with that dataset and how it compares to the predictions of the model-based approach proposed in that paper. In other words, would it be possible for the authors provide a wider assessment of their

© European Molecular Biology Organization

proposed framework?

3. Biological replication is a major concern. On page 5, the authors state that "To minimize biological variability, all samples were withdrawn from one bioreactor". Essentially, the authors present data from a single biological replicate (sampled in triplicate). While they refer to these as "biological replicates" throughout the manuscript, they really are just technical replicates, since they came from the same batch culture. Due to this limitation, it is difficult to assess whether these results are reproducible. Capturing the biological variability from these datasets could be crucial for discriminating statistically significant (and reproducible) observations from spurious ones.

4. Yeast grows about 1.5 times faster on glucose+glutamine compared to glucose+proline, so how much of the observed change in transcript abundance can be attributed to global non-specific changes in the cell due to changing growth rates (pages 5-6)? Can this be easily delineated from specific responses? How could one tell apart specific from non-specific?

5. For the parallel network motif (TORC1-independent), the authors state that the dynamic dependency should be high in the up and down shifts but random upon rapamycin treatment (page 8). I struggle to understand this. If a metabolite acts independent of TORC1, how does TORC1 inactivation affect its dynamic dependency causing it to become random? Based on the description on DD in the paper, this does not seem logical and needs to be clearly explained in the text.

6. Many figures are illegible. Trends in metabolite data are difficult to read (Figure 3A-B). Very hard to distinguish filled and open circles in Figure 4C. The scatterplot inset in Figure 4D is completely unreadable. Supplementary Figures 3 and 5 are especially difficult to decipher. In general, the figures have room for improvement.

7. Proteomic data is included in the methods and motivation but absent from the author's analysis. What did the authors learn/conclude from the proteomic measurements?

8. The authors report that "the cellular states at the start and end points of up- and downshifts were in close vicinity" (Page 5) based on PCA analysis of 909 metabolic enzymes. Yet, Supplementary Figure 4 (which includes all measured transcripts), does not support this association. Is it correct to call these similar "cellular states" when only the metabolic enzymes support the conclusion?

9. The "Probabilistic Assignment to Network Motifs" section is difficult to read and assess. In particular the subsection on "Bayesian inference framework" (Page 19) is difficult to interpret, specifically calculation of the likelihood value. Perhaps the authors can provide an equation and/or provide an example calculation in Figure 4 (in context of the T6P example)?

Minor issues:

1. Page 5 line 26 the authors state "Altogether 3202, 1585 and 2104 transcript abundances changed by more than 2 fold". Please provide more details about what you mean by this. Is the two fold change the total between each 2 consecutive time points, between any 2 time points, between the first and last time point?

2. What percentage of the 909 metabolic genes showed statistically significant changes in transcript abundance (2-fold)?

3. What is 'p' in the equation in Figure 4B? This information is not available in the figure legend.

4. Some measurement of cluster dispersion in Figure 2C would be helpful (e.g., standard deviation).

5. Providing graphical labels in Figure 1A (filled and open circles, triangles) would be helpful, rather than simply text.

6. Relevance of Figures 2A-B (especially given result in Figure 1C) is unclear

7. For any approaches that integrate prior knowledge into a computational framework the quality

and validity of results obtained can be highly dependent on the quality of this prior knowledge and may little to do with the computational framework in itself. Thus the authors should discuss this and educate the reader of much of an impact this can have on the system. The author could also try incorporating some "noise" into the prior information used to see how robust predictions are to false positives/negatives in prior knowledge. This might guide those trying to implement this framework in future for less well characterized networks.

8. In general, the authors should discuss some of the limitations of their proposed approach.

9. Were any of the metabolites measured by LC-MS/MS also measured by FIA-QTOF-MS? How was the agreement between the two methods? Can it provide an estimate of how reliable the FIA-QTOF-MS data is as input for this analysis?

10. While the proposed framework is generic enough to integrate protein abundances, this paper does not in fact actually use this data from the modeling perspective, so we have no idea how predictive or useful this model will be for integrating proteomics data which is much more difficult to work with than transcript abundances. The authors should make this clear in the manuscript and maybe de-emphasize the use of protein abundance in other parts of the manuscript (e.g. the abstract).

11. For illustrative purposes, could the authors consider alternative network motifs such as a hybrid between upstream and parallel, where a metabolite acts on some genes through TOR and on other genes in parallel with TOR?

12. Figure legend for 4B should include a description of all the abbreviations used.

13. On page 9 and 10, the authors conclude that TORC1 inhibits Hom3 and/or Hom2. However, I struggle to see how their data supports this. If aspartate accumulates (i.e., its conversion is inhibited) upon inhibition of TORC1 by rapamycin, that would mean enzymes involved in its conversion (Hom 3/2) are also inhibited when TORC1 is inhibited. So it is not clear how this is equated to TORC1 inhibition of these proteins (as opposed to activation).

14. On page 14 authors should provide a reference for OD to dry weight conversion coefficient provided.

15. Page 15 line 4 SRM is used before it is defined.

16. On page 17, why weren't the transcript abundances standardized like metabolite intensities prior to PCA analysis.

<u>Typos:</u>
Page 4 line 13: "resembles that to less-preferred" should be "of less preferred".
Page 5 line3: "and by glutamine depletion" should be "or by..."
Page 7 line 17: "metabolite changes common" missing word "in"?
Page 14 line 8: "also referred as sample time 0" missing word "to"?
Figure 2A legend "for every pair of transcripts" should this be "for each transcript"?

Reviewer #3:

The manuscript describes the integration of data from transcriptomic, proteomics and metabolomics measurements to analyze the regulation of nitrogen metabolism by the Target of Rapamycin complex 1 (TORC1) in S. cerevisiae. A role for S. cerevisiae TORC1 in regulating growth in response to changes in nitrogen quantity or quality is well established and has been demonstrated by genetic, biochemical, transcriptomic and proteomics analyses. The present manuscript presents a systematic approach that also combines metabolomic measurements. The authors' goal is to "...infer causal relationships between metabolism, signaling and gene regulation" (see Abstract, page 2). This goal is only partly achieved as correlation between changes in the concentration of certain metabolites and TORC1-dependnet transcription or protein modification changes does not

necessarily imply a cause and effect relationship.
The present study uses bioreactor batch culture and employs systematic measurements over time of a large number of parameters (cell size, mRNA level, protein level, intracellular metabolite levels, Sch9 phosphorylation as readout for TORC1 activity), leading to a comprehensive description of the response to changes in nitrogen availability via TORC1 signaling. The data presented is of high quality. The experiments are carefully designed and executed. The strength of the manuscript lies in metabolomics measurements, which add another level of complexity that has been poorly described before. Nevertheless, my main criticism is that while the present study confirms previous findings (i.e. that glutamine acts to activate TORC1), it does not prove novel TORC1 activation signals or downstream outputs. Although it identifies metabolites as putative downstream or upstream metabolites to TORC1, it does not show a proof of concept (correlation being an insufficient proof for cause-effect relationship). A potentially very interesting finding is that AICAR acts upstream of TORC1, while IMP, the next intermediate in purine synthesis, acts downstream of TOR1. As I indicate below, relatively simple experiments can address the possibility that AICAR increase and TORC1 activity are linked by a cause and effect relationship. Experimental validation of the proposed model should be a pre-requisite for publication.

Major points

In order to demonstrate that AICAR is a signaling intermediate that causes the up regulation of TORC1 signaling, the authors should show that accumulation of AICAR can lead to activation of TORC1. Thus, for example, the authors can explore the effect of mutating ade16 and/or ade17, the enzymes that catalyze the conversion of AICAR to IMP, on the activity of TORC1. Alternatively, AICAR and IMP may be artificially introduced into the cells and their effect directly monitored.

1st Revision - authors' response 20 January 2015

(see next page)

Editor:

Thank you again for submitting your work to Molecular Systems Biology. We have now heard back from the three referees who agreed to evaluate your manuscript. As you will see from the reports below, the referees acknowledge that the presented approach is potentially interesting. However, they raise a series of concerns, which should be carefully addressed in a revision of the manuscript.

*Without repeating all the comments listed below, some of the more **fundamental issues** are the following:*
- Follow-up experimentation validating the model's predictions of novel metabolic signals upstream/downstream TORC is required. Reviewer #3 provides constructive suggestions regarding this point.
- Reviewer #1 raises concerns on the choice of motifs and potential issues with motif identifiability.
- The broader applicability of the presented framework should be discussed.
Moreover, the reviewers refer to the need to extend the description of the methodology.

Please refer to the responses to reviewers below that address all fundamental issues listed above.

*On a more editorial level, we would like to ask you to **provide individual files for the Supplementary Tables and Supplementary Datasets**. Additionally, we would like to ask you to include the links and accession numbers to all datasets (i.e. microarray data) in the "Data Availability" section of your manuscript and to **provide the code used for the model-based analysis**.*

As requested, we included the section Data Availability after the Materials and Methods section, and links and accession numbers to the gene expression (http://www.ncbi.nlm.nih.gov/geo/query/acc.cgi?token=qxircgmwxtgvfid&acc=GSE54852) and protein levels datasets (we have kept the same information in the corresponding sub-sections of the Materials and Methods (“Determination of mRNA levels” and “Determination of protein levels”) as before). The code used for model-based analysis (including documentation and test examples) is now available as Supplementary Data 1.

Reviewer #1:

The authors present a novel inference method based on Bayesian modeling to predict the how metabolites regulate gene expression. The method is based on choosing the 'motif' that best explains the dynamics of the global transcriptional responses.

The method is applied to a new and very interesting by itself data set that combines dynamic (time-series) metabolomic, transcriptomic and proteomic data under three different conditions using wild-type S.cerevisiae cells: two to capture metabolic shifts between poor N-source (proline) and rich N-source (glutamine), and the third to show the effect inhibition of TORC1 by Rapamycin. High changes at the transcriptional level were identified, and to study the possible drivers of these changes via TORC1 the inference model was applied using four different motifs (no-effect, acting upstream, acting downstream, or in parallel with signalling pathways of interest) that were assembled based on prior-knowledge in the literature. The authors conclude from the model and the metabolomics experiments that most of the metabolites were unrelated to the transcriptomics changes, 7 to be downstream of TORC1, 7 parallel and 6 upstream TORC1. They provide partially indirect validation (by testing against literature) and propose new leads for further investigation.

This paper presents a novel approach to an important current problem in the field of systems biology: how to link different cellular processes (metabolism, signalling and gene regulation). As authors state in the introduction, there are only few methods for this, and in particular in the context of dynamics, which is expected to be a key aspect in these processes and their interactions. Hence, methods such as this are highly needed and can have a potential important impact in the community.

The method sounds solid from the mathematical and statistical point of view, and some robustness tests have been done. However, more detail would be useful (as outlined below).

The choice of the case study to illustrate their method is a very adequate one: TOR is a well-known regulator of metabolism, and the knowledge of its role is yeast has been widely study. In addition, yeast is a good model system to perform different 'omics' experiments with expected good quality. Accordingly, the experimental set-up is well defined and the different time-points for the measurements allow to capture the dynamics that are characteristic of nutritional changes at the level of protein and metabolic networks.

While we consider this a potentially important contribution to the field, we feel the following points should be addressed to accurately assess its value.

Major revisions:

***1.1** The networks motifs were selected based on prior-knowledge, but is not clear why authors have chosen those and then not taken into account other types of motifs such as TOR -> Met -> Genes. Also, the "downstream" network (fig. 4a) is unclear: there is an arrow TOR->genes, and one TOR-> MET, but why not MET-> genes? (as is in e.g. the parallel case)? Even if there is compelling evidence for some motifs over others, it shouldn't be computationally difficult to explore further motifs.*

A related point is that of identifiability. Some of the motifs may not be distinguishable from each other - for example, the TOR- > MET- > Genes and the (TOR- > MET & TOR- > Genes) versions may not be distinguishable?

Also what happens if some metabolite has multiple connections in this network, both up- and downstream of TOR?

In general, some more explanation on the choice of motifs and their potential identifiability would be important. Authors could include some analyses to asses this.

We thank the reviewer for addressing these important questions. We have now provided detailed explanations of the rationale for network motif selection and of the relations between network motifs as shown in Figure 4A and network wiring diagrams as a new Supplementary Text 1. Briefly, the logic of our study and the biological knowledge on mechanisms of TORC1 signaling exclude all but the four 'prototypic' motifs shown in Figure 4A among all possible outcomes of the experimental and computational analysis; they also exclude the TOR->Met->Genes wiring pointed out by the reviewer. In addition, while our four 'prototypic' motifs are structurally identifiable, this does not imply that the wiring diagram of a motif (as shown in Fig. 4A) is unique. Overall, we agree that identifiability is a critical issue, and in addition to detailed explanations in Supplementary Text 1, we have added statements to the Discussion to explicitly point out corresponding limitations of the concept (e.g. that multiple interactions of a metabolite on the relevant time-scales cannot be distinguished).

1.2 *Some of the hypothesized candidates were validated with references from the literature. We appreciate that to perform biological validations of the candidates may not be trivial. That said, some experimental validation assessing for instance the post-translational regulation of the downstream candidates of TORC1 would complement the existing validation and provide further evidence of the usefulness of the method. Are any of these possible? We do not consider this a requirement for publication, but certainly would strengthen the paper.*

Generally we agree with the reviewer, although empirical validation of a downstream TOR target could not actually validate the method. Please note that upon submission of this manuscript, essentially no enzyme targets of TORC1 where really known in yeast. One of our key contributions is to provide first evidence for several such enzymes that are likely to be subject to TORC1-dependent post-transcriptional regulation.

To address the issue of TORC1 downstream targets more comprehensively, we instead conducted a more systematic study with dynamic phosphoproteomics data for the identical three experiments. Combining these phoshoproteomics data with the here reported metabolite data, we could infer several enzymes whose phosphorylation state changes consistently with increased/decreased TORC1 signaling; thereby validating three of the here predicted downstream targets Amd1, Hom3 and Tsl1. Since these experiments and their analysis go well beyond the scope of the present manuscript, they were included in a separate manuscript that has now been accepted for publication with Science Signaling (document

made available to the reviewer). We hope that the reviewer and editor will agree that this is at least as meaningful as testing a single case.

1.3 *From the (few) existing approaches to link metabolism to regulatory processes, many of them use metabolic (and sometimes regulatory and/or signaling) networks as a basis. In fact, the authors have done important contributions in this area (including recently Oliveira et al. MSB 2012). Can the approach presented here be combined with those methods? At least it should be discussed the potential relationships.*

Here, we did not use metabolic networks for the inference (but for the mapping of inferred metabolite characteristics as in Fig. 5), but it is in principle possible to incorporate such networks either as prior knowledge (as for the experimentally determine TF-gene interactions used for the representation of TOR genes) or as a more refined causal model (e.g., similar to Fig. 4B: any change in a metabolite concentration should be preceded by changes in educt concentration(s) in the corresponding reaction(s)). We emphasized such possibilities in the Discussion (last paragraph).

1.4 *Although the problem is well contextualised, the methods are not explained in detail, and some aspects are not clear. For instance, it is not clear if the proteomics data-set that was mentioned in the introduction was actually used in the inference method. Or what is the exact nature of the models: in Fig 4B a general ODE equation is described, but what is the exact set of equations (for each of the motifs)?*

We have addressed the explanation of methods by revising the Methods section (see also reply to 2.9). Specifically, the proteomics data set provided an important input to the inference method by (i) showing that proteome changes at the fast time-scales considered are not to be anticipated, and (ii) that this holds for all perturbations applied to the system.

The ODE in Fig. 4B illustrates a fundamental assumption of the method (the models employed are statistical in nature and described in the Methods section), namely that there exists a functional relation between the concentration of a metabolite and the rate of change of gene expression (directly or indirectly via e.g., TOR signaling). The inference of causal relations exploits this functional relation via the dynamic dependency (time-shift between metabolite profiles and derivative of transcript profiles); key to the approach is that no further specification of the function itself is required (now explained in revised legend to Fig. 4B).

Minor revisions:

# Related to the above point on clarity, some points are present in the methods, but the connection from main text is not clear. For example, it is stated 'For every metabolite [...] and then determined the fraction of genes known to be targeted by TORC1-dependent transcription factors". It is only when one goes to

the the materials and methods that is explained how is this done ,the use of the YEASTRACT database, etc - pointers to materials and method would help here.

We added corresponding pointers and clarifications in the revised text (section ‘A computational method …’).

# Authors should make their code available

The code (including documentation and working examples) is available as Supplementary Data 1.

# Authors could elaborate on how this could be applied in other contexts - is this applicable say in human and in particular disease biology?

The approach is applicable in other contexts provided that significant prior knowledge exists (e.g., a relevant part of the transcriptional network controlled by the signaling pathway of interest), and that it is possible to perturb the system by drugs equivalent to rapamycin in our study (in this respect, a transfer of the approach to mammalian TOR signaling would be direct). We have added corresponding text to the Discussion.

Suggestions:
# A large part of the manuscript is devoted to describe the data (both in text and in figures). We would suggest to shorten this part (and move details to the supplements), and rather explain in more detail the method and the analyses performed with it, as stated above: these are the key novel aspect of this paper, and the harder to grasp, in our opinion. For example, Figure 2 is used to support the statement that the Rapamycin treatment condition is similar to N-downshift condition (glutamine depletion). Maybe this figure could be part of the supplementary materials and give more emphasis to the methods.

We acknowledge the desire of the reviewer(s) to see more details on the computational method, and we have now extended the Methods description and we provide a new Supplementary Text 1. With respect to Figure 2, it appears critical to us to show detailed data because our approach requires (i) careful selection of time-scales, and (ii) consistent detailed system responses for pairwise computations of dependencies that well extend beyond similarities of shifts. We therefore prefer to leave the rather short description of the experimental data (and figures) as they are.

Reviewer #2:

Summary. Cells respond to changing environmental conditions through the integrated use of highly complex signaling, transcriptional and metabolic networks. A major challenge of systems biology is gaining a better understanding of how these networks are interconnected and deconvoluting the sequence of events that results in specific cellular responses. Oliveira and Dimopoulos et al. report construction of a probabilistic framework to integrate dynamic gene expression and metabolite measurements in S. cerevisiae, which they use to infer causal influences that act upstream and downstream of TORC1 in response to alternative nitrogen sources. Their framework integrates time-course data of metabolite, (protein -not compelling) and transcript abundance to determine causal relationships by taking advantage of prior knowledge.

In general, the approach seems sound and the analysis/statistical framework is rigorous, but there are significant concerns with regard to experimentation. Also, for publication in a high profile journal like MSB, the authors need to provide validations for key model predictions.

Major issues

2.1. *While the model predicts several known (or strongly hypothesized) features of TORC1 signaling (e.g., role of glutamine and TORC1-independence of tryptophan synthesis) and the authors provide interesting speculations based on the model's predictions (e.g., TORC1-dependent (de)phosphorylation of Hom3/Hom2 and Tsl1/Tps3, and TORC1 role in AICAR->IMP conversion), it is unclear what has been learned about TORC1 signaling (other than model-derived speculation). To increase biological significance of the work, the authors should provide compelling evidence and validation for other predictions. For publication in a top-tier journal like MSB, such validations would be critical.*

We would like to stress that the main merit of this manuscript is, besides a highly reproducible, quantitative and dynamic data set, a new approach to infer causality of signals from metabolism to transcription. The field is dearly in need of such methods that integrate different data sets for the purpose of hypotheses generation in complex situations where intuitive reasoning breaks down. For metabolomics data, hardly any such methods exist. Having said that, we do agree of course that experimental validation is important. Instead of choosing a particular example as a follow-up, we opted for a more systematic approach using dynamic phosphoproteomics data that provided additional evidence for phosphoregulation of Amd1, Hom3 and Tsl1 in response to TORC1 signaling. Since these experiments and their analysis go well beyond the scope of the present manuscript, they were included in a separate manuscript that has now been accepted for publication with Science Signaling (document made available to the reviewer). We hope that the reviewer and editor will agree that this is at least as meaningful as testing a single case (see also point 1.2 of reviewer 1).

2.2. *I presume the proposed framework could be used with the Bacillus subtilis data generated by this group in Buescher et al. 2012. It would be interesting to see how the proposed framework performs with*

that dataset and how it compares to the predictions of the model-based approach proposed in that paper. In other words, would it be possible for the authors provide a wider assessment of their proposed framework?

While this is an interesting idea, as emphasized in Abstract and Discussion, our approach requires a co-design of experiments such that the inference method can be applied (in its present form). Specifically, inference relies on (i) experimental data for input and output quantities (metabolites and gene expression in the present case) and (ii) the ability to not only perturb measured input quantities (external metabolite concentrations) but also to set equivalent perturbations on unmeasured internal quantities (here: inhibition of TORC1 activity by rapamycin) in order to infer relations between inputs, outputs, and signaling. Because condition (ii) is not fulfilled by the Buescher et al. 2012 data set, we cannot provide such an assessment here. We have added corresponding clarifications to the Discussion.

2.3. *Biological replication is a major concern. On page 5, the authors state that "To minimize biological variability, all samples were withdrawn from one bioreactor". Essentially, the authors present data from a single biological replicate (sampled in triplicate). While they refer to these as "biological replicates" throughout the manuscript, they really are just technical replicates, since they came from the same batch culture. Due to this limitation, it is difficult to assess whether these results are reproducible. Capturing the biological variability from these datasets could be crucial for discriminating statistically significant (and reproducible) observations from spurious ones.*

We apologize for this misunderstanding. Three independent replicate experiments were done for each dynamic shift. For EACH of the replicates, all aliquots for different analysis were withdrawn from the same bioreactor experiment. Relative to what is normally reported in multiple omics studies, we believe that our design is extraordinarily rigorous in terms of minimizing biological variability. We have now clarified this in the text (page 5 “To minimize biological variability, all samples for the different omics were withdrawn from one bioreactor culture in three independent biological replicates, at a temporal range […]”), as well as in the Materials and Methods.

2.4. *Yeast grows about 1.5 times faster on glucose+glutamine compared to glucose+proline, so how much of the observed change in transcript abundance can be attributed to global non-specific changes in the cell due to changing growth rates (pages 5-6)? Can this be easily delineated from specific responses? How could one tell apart specific from non-specific?*

For a steady state analysis, this difference in growth rate would have a significant impact. In our case, however, we monitor the immediate dynamic response, to environmental perturbations. The vast majority of the data are from a phase where the growth rate has not noticeably changed yet (we have not observed changes in growth rate in the hour following the shifts relative to the observed maximum growth rate during the pre-shift exponential phases).

2.5. *For the parallel network motif (TORC1-independent), the authors state that the dynamic dependency should be high in the up and down shifts but random upon rapamycin treatment (page 8). I struggle to understand this. If a metabolite acts independent of TORC1, how does TORC1 inactivation affect its dynamic dependency causing it to become random? Based on the description on DD in the paper, this does not seem logical and needs to be clearly explained in the text.*

The dynamic dependency provides a measure for how well changes in metabolite concentrations correlate with subsequent responses in gene expression rates, thus evaluating the likelihood of a causal relation between a metabolic and gene expression dynamics. For the parallel network motif, addition of rapamycin will lead to gene expression response by TORC1, but not by the specific metabolite (because at the time-scales considered, the metabolite concentration should not change unless the metabolite is at the same time downstream of TORC1; see response to 1.1. for the case of overlapping motifs). In that case, the metabolite concentration would not be predictive of gene expression changes, implying a random dynamic dependency.

We have added clarifying statements in the main text for the parallel network motif. In addition, for better explanation of the detailed rationale of motif definitions and inference based on measured features, we now provide an extended Supplementary Text 1 (see also response to 1.1).

2.6. *Many figures are illegible. Trends in metabolite data are difficult to read (Figure 3A-B). Very hard to distinguish filled and open circles in Figure 4C. The scatterplot inset in Figure 4D is completely unreadable. Supplementary Figures 3 and 5 are especially difficult to decipher. In general, the figures have room for improvement.*

All figures were re-edited to improve readability. In particular, font sizes were increased and all main text figures were modified to more or less extent to address other reviewers' points and/or facilitate interpretation. The "problem" with Figure 3 was most likely simply a poor quality in the pdf version for which we apologize. Three lines of connected data points for selected metabolites clearly show whether or not a consistent response occurs; i.e. opposite direction of upshift (red) vs chemical/nutritional downshift (green, blue). Absolute values are not relevant for this analysis, although the data are of course listed in the supplement. The scatter plot of Figure 4D was indeed not well presented and has removed. Similarly, we restructured Figure 5 for clarity.

2.7. *Proteomic data is included in the methods and motivation but absent from the author's analysis. What did the authors learn/conclude from the proteomic measurements?*

As stated in the text, the 20 TOR signaling proteins whose abundance we monitored did not change during the three shifts, rendering their analysis obsolete (last sentence 3rd paragraph of the results). We learned that the concentration of regulatory proteins in the TORC1 cascade does not contribute significantly to the responses, which was an important prerequisite for the inference methods (see response to 1.4).

2.8. *The authors report that "the cellular states at the start and end points of up- and downshifts were in close vicinity" (Page 5) based on PCA analysis of 909 metabolic enzymes. Yet, Supplementary Figure 4 (which includes all measured transcripts), does not support this association. Is it correct to call these similar "cellular states" when only the metabolic enzymes support the conclusion?*

At the suggestion of the reviewer (see also point 16) we have now pre-processed the transcript abundances for PCA differently (mean-centered and scaled to unit variance), and observed that either when considering only metabolic genes or all genes, the start and end-points of up- and downshift were in close vicinity. Therefore we keep our original phrasing.

2.9. *The "Probabilistic Assignment to Network Motifs" section is difficult to read and assess. In particular the subsection on "Bayesian inference framework" (Page 19) is difficult to interpret, specifically calculation of the likelihood value. Perhaps the authors can provide an equation and/or provide an example calculation in Figure 4 (in context of the T6P example)?*

We rewrote the "Probabilistic Assignment to Network Motifs" section and included a clear description of all the steps of the computational method as well as the corresponding equations for the Bayesian inference framework.

Minor issues

1. Page 5 line 26 the authors state "Altogether 3202, 1585 and 2104 transcript abundances changed by more than 2 fold". Please provide more details about what you mean by this. Is the two fold change the total between each 2 consecutive time points, between any 2 time points, between the first and last time point?

We thank the reviewer for pointing out the omitted definition. Transcript fold changes refer to the maximum observed fold-change across each transcript time-course (maximum intensity divided by the minimum throughout the time course). This information has now been added on p. 5. We have also corrected a typo in the number of transcripts changing by more than two-fold in the N-upshift (3203 instead of 3202).

2. What percentage of the 909 metabolic genes showed statistically significant changes in transcript abundance (2-fold)?

Out of the 909 metabolic genes, 519 (N-upshift), 293 (N-downshift) and 376 (rapamycin) transcripts change by more than two-fold relative to the steady-state sample (as per definition in the previous point). We would like to note that we assess changes based on the maximum fold-change across the time-course of the experiment in which all transcript time points were measured, and therefore the

numbers presented in this part of the text are not statistically evaluated based on triplicates (triplicates are only taken into account in the statistical dependency model). Therefore, we did not include the above in the main text.

3. What is 'p' in the equation in Figure 4B? This information is not available in the figure legend.

‘p’ refers to unknown model parameters; we have augmented the caption to explain all symbols and notation.

4. Some measurement of cluster dispersion in Figure 2C would be helpful (e.g., standard deviation).

Figure 2C now shows cluster means as well as their standard deviation as a measure of dispersion.

5. Providing graphical labels in Figure 1A (filled and open circles, triangles) would be helpful, rather than simply text.

Graphical labels have now been included in Figure 1A, by the y-axis label.

6. Relevance of Figures 2A-B (especially given result in Figure 1C) is unclear

Fig. 1C shows consistency of the shift experiments (especially: nutrient down-shift and rapamycin) at the level of principal components, capturing about 60% of the dataset variance. For the inference method, which relies on correlations between all individual metabolite-transcript pairs, however, it is essential to show that the shifts are also consistent at the level of the individual measured entities, for which Fig. 2A-B shows the evidence. Fig 2A provides a clear indication that a large fraction of transcripts are positively or negatively correlated across the shifts, a result that complements the information for the clustering (Fig. 2C). Fig. 2B provides indicators of the timing of responses at the level of individual transcripts. Together, the different panels of Fig. 2 provide complementary information showing that a large fraction of responses are positively/negatively correlated across the shifts, while a few responses are specific from each shift. These observations are essential to on one side verify the complementary of the shifts, and on the other side depict the specific transcriptional responses of each shift. We have added a corresponding justification to the ‘Extensive dynamic rewiring …’ section.

7. For any approaches that integrate prior knowledge into a computational framework the quality and validity of results obtained can be highly dependent on the quality of this prior knowledge and may little to do with the computational framework in itself. Thus the authors should discuss this and educate the reader of much of an impact this can have on the system. The author could also try incorporating some "noise" into the prior information used to see how robust predictions are to false positives/negatives in

prior knowledge. This might guide those trying to implement this framework in future for less well characterized networks.

Here, we integrated prior knowledge of the following type: (i) general functional relations between entities such as the existence of an unknown metabolite serving as an input to TORC1 signaling, and (ii) specific prior knowledge in terms of known transcription factors that are directly controlled by TORC1 as well as these transcription factors' target genes. Quality issues with prior knowledge would affect (ii), e.g. in the form of unknown transcriptional targets of TORC1-controlled TFs. We employed only experimentally validated TORC1-TF (direct biochemical evidence that the TF is regulated via TORC1) and TF-gene direct and documented interactions from the YEASTRACT database, which are expected to incorporate relatively few false negatives. The general issue is certainly important (especially with respect to biased sampling of the real transcriptional network), and we included corresponding statements in the Discussion.

8. In general, the authors should discuss some of the limitations of their proposed approach.

We have extended the Discussion correspondingly, focusing on (i) required co-design of experiments and inference approach, (ii) effects of uncertain prior knowledge, and (iii) mixed or non-identifiable motifs.

9. Were any of the metabolites measured by LC-MS/MS also measured by FIA-QTOF-MS? How was the agreement between the two methods? Can it provide an estimate of how reliable the FIA-QTOF-MS data is as input for this analysis?

Given that LC-MS/MS is a targeted measurement of about 30 absolute concentrations and FIA-QTOF-MS an untargeted general scan of the metabolome with only relative information, it is quite an achievement that the two measurements generally generate consistent results. In the new Supplementary Figure 6 we now provide a correlation analysis (part A) and compare also for selected important examples the time-dependent measurements (new sentence on page 15 "The overall performance of the untargeted method was compared against the LC-MS/MS measurements for the common metabolites measured in both platforms (Supplementary Figure 6), and generally the Spearman correlation coefficient was high for those metabolites changing above two-fold".). FIA-QTOF data hence is generally suitable for our computational approach, although when given the choice one would prefer to rely on data coming from targeted LC-MS/MS metabolomics. The strength of FIA-QTOF is the broad overview that is simply beyond most targeted methods.

10. While the proposed framework is generic enough to integrate protein abundances, this paper does not in fact actually use this data from the modeling perspective, so we have no idea how predictive or useful this model will be for integrating proteomics data which is much more difficult to work with than transcript abundances. The authors should make this clear in the manuscript and maybe de-emphasize the use of protein abundance in other parts of the manuscript (e.g. the abstract).

We agree with the reviewer's rationale and we have carefully re-analyzed all relevant text. As discussed above, the proteomics data was critical for formulating the specific inference approach (because protein dynamics could be neglected) and also in the abstract we do not make statements on other use of proteomics data. We have now rewritten the final paragraph of the Discussion to make clear what type of information is needed to apply the framework, besides the data it is mainly a network topology. There is no question about it, the framework is entirely generic and very well equipped to work with protein data, only in our case they did not change. From the perspective of the framework, protein data are as difficult/simple to work with as transcript data, but obviously the former are more difficult to obtain at the same level of comprehensiveness and accuracy as the latter.

11. For illustrative purposes, could the authors consider alternative network motifs such as a hybrid between upstream and parallel, where a metabolite acts on some genes through TOR and on other genes in parallel with TOR?

Please refer to our answer to 1.1 (we have added a corresponding extension to the revised text).

12. Figure legend for 4B should include a description of all the abbreviations used.

The legend has been augmented.

13. On page 9 and 10, the authors conclude that TORC1 inhibits Hom3 and/or Hom2. However, I struggle to see how their data supports this. If aspartate accumulates (i.e., its conversion is inhibited) upon inhibition of TORC1 by rapamycin, that would mean enzymes involved in its conversion (Hom 3/2) are also inhibited when TORC1 is inhibited. So it is not clear how this is equated to TORC1 inhibition of these proteins (as opposed to activation).

The above logic is a misinterpretation of our statements, since we never claimed that TORC1 inhibits Hom3. The argument is that reduced TORC1 activity leads to an inhibition of Hom3, hence we call this TORC1-dependent inhibition of Hom3. The influence can be mediated via one or more downstream kinases, and in fact in our phosphoproteomics paper we identify the Atg1 kinase as the likely mediator. Still, the regulation is TOR dependent. Nevertheless, we thank the reviewer for the comment. To avoid confusion, we replaced on page 10 the word "inhibition" by "regulation".

14. On page 14 authors should provide a reference for OD to dry weight conversion coefficient provided.

The OD to dry weight conversion coefficient was determined by us in the present study, for our strain and conditions. We mistakenly used the expression "previously" when we actually meant "before we started the bioreactor cultures". We have now adjusted the sentence on page 14 accordingly, by replacing "previously" with "initially".

15. Page 15 line 4 SRM is used before it is defined.

The definition is now provided at first use.

16. On page 17, why weren't the transcript abundances standardized like metabolite intensities prior to PCA analysis.

There was no particular reason for the different standardization other than the broader range of metabolite measurements calling for a scaling to unit variance before the PCA. Nevertheless, at the reviewer's suggestion, we have now pre-processed both transcripts and metabolite intensities in the same manner, that is, the data was mean-centered and scaled to unit variance. This resulted in a slight modified version of Figures 1C and Supplementary Figure 4. The corresponding text in the Materials and Methods section was modified accordingly.

Typos
Page 4 line 13: "resembles that to less-preferred" should be "of less preferred".

The text is revised: 'The response to rapamycin-induced inhibition of the TOR complex 1 (TORC1) resembles the response to less-preferred N-sources …'.

Page 5 line3: "and by glutamine depletion" should be "or by..."

Changed.

Page 7 line 17: "metabolite changes common" missing word "in"?

Changed.

Page 14 line 8: "also referred as sample time 0" missing word "to"?

Changed.

Figure 2A legend "for every pair of transcripts" should this be "for each transcript"?

The notation 'pair of transcripts' is correct (correlation between any two transcripts).

Reviewer #3:

The manuscript describes the integration of data from transcriptomic, proteomics and metabolomics measurements to analyze the regulation of nitrogen metabolism by the Target of Rapamycin complex 1 (TORC1) in S. cerevisiae. A role for S. cerevisiae TORC1 in regulating growth in response to changes in nitrogen quantity or quality is well established and has been demonstrated by genetic, biochemical, transcriptomic and proteomics analyses. The present manuscript presents a systematic approach that also combines metabolomic measurements. The authors' goal is to "...infer causal relationships between metabolism, signaling and gene regulation" (see Abstract, page 2). This goal is only partly achieved as correlation between changes in the concentration of certain metabolites and TORC1-dependnet transcription or protein modification changes does not necessarily imply a cause and effect relationship.

The present study uses bioreactor batch culture and employs systematic measurements over time of a large number of parameters (cell size, mRNA level, protein level, intracellular metabolite levels, Sch9 phosphorylation as readout for TORC1 activity), leading to a comprehensive description of the response to changes in nitrogen availability via TORC1 signaling. The data presented is of high quality. The experiments are carefully designed and executed. The strength of the manuscript lies in metabolomics measurements, which add another level of complexity that has been poorly described before. Nevertheless, my main criticism is that while the present study confirms previous findings (i.e. that glutamine acts to activate TORC1), it does not prove novel TORC1 activation signals or downstream outputs. Although it identifies metabolites as putative downstream or upstream metabolites to TORC1, it does not show a proof of concept (correlation being an insufficient proof for cause-effect relationship). A potentially very interesting finding is that AICAR acts upstream of TORC1, while IMP, the next intermediate in purine synthesis, acts downstream of TOR1. As I indicate below, relatively simple experiments can address the possibility that AICAR increase and TORC1 activity are linked by a cause and effect relationship. Experimental validation of the proposed model should be a pre-requisite for publication.

Major points

3.1 *In order to demonstrate that AICAR is a signaling intermediate that causes the up regulation of TORC1 signaling, the authors should show that accumulation of AICAR can lead to activation of TORC1. Thus, for example, the authors can explore the effect of mutating ade16 and/or ade17, the enzymes that catalyze the conversion of AICAR to IMP, on the activity of TORC1. Alternatively, AICAR and IMP may be artificially introduced into the cells and their effect directly monitored.*

We very much appreciate the suggestions of the reviewer as these signals are also our long range goal. We therefore performed the suggested experiments. Unfortunately, these experiments were neither “relatively simple” nor conclusive because there is no specific way of increasing intracellular AICAR concentrations. AICAR itself is toxic, hence cannot be added externally, and IMP was not taken up efficiently. Manipulation of intracellular AICAR levels through ade16 and ade17 mutations led to strong pleiotrophic effects with unclear requirements of medium components. We also tried to degrade the

Ade proteins, but the dynamics were too slow to be effective. Overall, the performed experiments do not reject our predictions, but they were simply inconclusive.

Since identification of the metabolite signals into regulation cascades in general and into TORC1 in particular is sort of the Holy Grail in the field, we believe that biochemical demonstration of such a mechanism for TOR is beyond the scope of a manuscript whose main merit is the method. Instead the results should be taken as what they are, well grounded hypotheses from a large dynamic data set. Biochemical demonstration of TORC1 modulation through AICAR or other predicted metabolites (directly or through upstream effectors) would rightfully earn a Nature paper. To keep with the idea of validation, we instead opted to look for the downstream of TORC1 targets as was discussed in point 1.2 of reviewer 1 and 2.1 of reviewer 2.

2nd Editorial Decision 24 February 2015

Thank you again for submitting your work to Molecular Systems Biology. We have now heard back from the referees who agreed to evaluate your manuscript. As you will see below, the reviewers are satisfied with the modifications made and only list a few remaining concerns which we would ask you to address in a revision of this work.

Reviewer #1:

We thank the authors for the revised version and their efforts to address our concerns.

We feel they have generally addressed adequately most of our concerns, as well as those of other reviewers.

In particular, we hare happy to read that there is experimental data in the just-accepted complementary publication that can serve as validation for the results of this paper, which was one of our (as well as from other reviewers) main concern.

Could the authors then refer to that data, and explain (in this manuscript), which part of that paper's data validate the results of this manuscript?

Our second main point was about the prior knowledge behind the motifs. As brought up by reviewer 2 as well (point 7), this can have an important effect on the results. Authors now discuss this in the paper, but we would suggest them (along the lines of the comment from Reviewer 2), to try to assess the potential effect of this uncertainty. For example, they could use a model to generate data with adequate level of noise and so on, and then apply their algorithm to a set of motifs (including the right one) and show that their method rightly identifies the right motif. Or also explore what happens if the right motif is not known, etc.

Minor point:
- Could authors report the percentage of variance explained by each PC in Figures 1C and 3C?. To make sure that not an important PC is not reported/represented. Specially in 3C, where the variance explained by the plotted PC1 and PC2 is relatively low.

Reviewer #2:

The authors have addressed nearly all of my concerns. I do have one lingering concern regarding the clarity of presentation vis-a-vis replicated experiments. I do not feel the modification made to the text has addressed the initial concern. How I interpret the current statement is that three independent samples were obtained from the same bioreactor. If samples were obtained from three independent chemostat runs (either done in parallel or sequentially), this should be clearly stated in the text. If not, then the initial concern has not been addressed.

2nd Revision - authors' response 19 March 2015

We thank you very much for your and the reviewersí constructive comments and suggestions for our revised manuscript.
With this revision, we have addressed all remaining concerns as well as the editorial points (please see the response to reviewers for details). In particular, our new, extensive computational studies on the robustness of predictions given uncertainties in prior biological knowledge (here: regarding the transcriptional network controlled by TORC1) showed that all the predictions shown and discussed in the manuscript are very stable (as detailed in the augmented text, data shown in new panels for Fig. 5). In addition, the analysis helped gaining intuition for the requirements of the approach, which may prove important for future applications; we wish to thank the reviewer for suggesting such an analysis.

Reviewer #1:

We thank the authors for the revised version and their efforts to address our concerns. We feel they have generally addressed adequately most of our concerns, as well as those of other reviewers. In particular, we are happy to read that there is experimental data in the just-accepted complementary publication that can serve as validation for the results of this paper, which was one of our (as well as from other reviewers) main concern. Could the authors then refer to that data, and explain (in this manuscript), which part of that paper's data validate the results of this manuscript?

We thank the reviewer for the appreciation of our efforts and the positive comments. To address the relation between the two studies, we have now added the following sentence at the end of the second paragraph of the discussion:

> 'Through a combination of the here reported metabolomics data with phosphoproteomics data, TORC1-dependent activation of T6P-synthase and Amd1 as well as inhibition of Hom3 were independently confirmed (Oliveira et al., Science Signalling, accepted for publication).'

Our second main point was about the prior knowledge behind the motifs. As brought up by reviewer 2 as well (point 7), this can have an important effect on the results. Authors now discuss this in the paper, but we would suggest them (along the lines of the comment from Reviewer 2), to try to assess the potential effect of this uncertainty. For example, they could use a model to generate data with adequate level of noise and so on, and then apply their algorithm to a set of motifs (including the right one) and show that their method rightly identifies the right motif. Or also explore what happens if the right motif is not known, etc.

We thank the reviewer for stressing the point of robustness of the method with respect to prior knowledge behind the motifs, and we addressed it by extensive computational analysis. Briefly, in addition to the definition of motifs (discussed in detail in Supplementary Text 1), the prior knowledge used are the assignments of transcription factors directly controlled by TORC1 and their respective associated genes. We investigated the effects of uncertainty in this prior knowledge to assess robustness of motif assignments (as well as the applicability of the method with respect to requirements on prior biological knowledge). The logic and main results of this analysis are summarized as follows in the revised first paragraph of the 'Computational assignment of metabolites to network motifs' section (along with corresponding changes in Fig. 5A, new panels Fig. 5B-D, and extended Methods section):

> 'To analyze the robustness of the assignments and how motif assignments depend on the prior biological knowledge on TORC1-controlled TFs and their controlled genes, we considered two scenarios: (i) prior knowledge is correct but limited - we know fewer than the 11 reported TFs directly controlled by TORC1 - and (ii) knowledge on TORC1-controlled TFs is incomplete, meaning that additional TFs could be directly controlled by TORC1. We investigated both scenarios for different numbers of TFs affected, and we refer to them as '-3 … -1 TFs' and '+1 … +3 TFs' scenarios, respectively (see Methods for details). Computing motif assignments for all metabolites and all perturbation scenarios revealed that the total error rate, defined as the average rate of

> assignments that differ from the unperturbed analysis, generally is below 30% (Figure 5B). Sufficient prior knowledge on real TORC1-TFs is important (red curves in Figure 5B), whereas additional unknown TFs have less effect (blue curves). If we expect errors in motif assignments, these will be false negatives (no motif is assigned although it should be, Figure 5C), rather than false positives (assignment of spurious motifs, Figure 5D). In particular, all assignment frequencies that exceed approximately 70% have practically zero false positive and false negative rates on average. For our original motif assignments shown in Figure 5A, we find generally high motif robustness as quantified by the motif recovery stability for the +3 TFs case. This scenario gives a lower bound in our application-relevant + TF scenarios; previous experimental analyses provide us with high confidence for the validity of the 11 known TORC1-TFs.'

Overall, we show that all the inference results discussed in the remainder of the section are robust to the extent of prior knowledge; in addition, the analysis gives an estimate of the required biological knowledge for applications in other biological contexts (see also added sentence in the Discussion to that effect).

Finally, while we agree with the reviewer that a complete in silico evaluation would be desirable for more extended benchmarking, unfortunately corresponding generative models exist only for gene regulatory networks. Designing corresponding models for the integrated (metabolism – signaling – transcriptional networks) system is feasible in principle, but it would require extensive model development and validation such as not to introduce additional uncertainties. We see this as outside the scope of the present analysis.

Minor point:
- Could authors report the percentage of variance explained by each PC in Figures 1C and 3C?. To make sure that not an important PC is not reported/represented. Specially in 3C, where the variance explained by the plotted PC1 and PC2 is relatively low.

The corresponding figure legends now provide the variance explained by the next-important component PC3. In both cases, the explained variance is less than one quarter of the value for PC1.

Reviewer #2:

The authors have addressed nearly all of my concerns. I do have one lingering concern regarding the clarity of presentation vis-a-vis replicated experiments. I do not feel the modification made to the text has addressed the initial concern. How I interpret the current statement is that three independent samples were obtained from the same bioreactor. If samples were obtained from three independent chemostat runs (either done in parallel or sequentially), this should be clearly stated in the text. If not, then the initial concern has not been addressed.

We apologize for remaining ambiguities in the statement on replicates. The first sentence of the Results section is now revised to clearly state that n=3 independent biological replicates per shift experiment were used:

> ‘To elucidate the dynamic interplay between the signaling, transcriptional, and metabolic networks controlling the cellular response to the quality of the external N-source, we subjected *S. cerevisiae* YSBN6 wild-type cells (Canelas et al, 2010) to nutritional up and down shifts *that were each done as fully independent biological triplicate experiments.*’

Acceptance letter 23 March 2015

Thank you again for sending us your revised manuscript. Reviewer #1 is now satisfied with the modifications made and thinks that the study is suitable for publication. Therefore I am pleased to inform you that your paper has been accepted for publication in Molecular Systems Biology.

Reviewer #1:

The authors have adequately addressed our final comments.

We appreciate that the in silico evaluation would be a significant amount of extra work, though we still encourage the authors to do this in a follow up study.

© European Molecular Biology Organization
